# Local Antigen Encounter Is Essential for Establishing Persistent CD8^+^ T-Cell Memory in the CNS

**DOI:** 10.3389/fimmu.2019.00351

**Published:** 2019-03-04

**Authors:** Amalie S. Schøller, Masja Fonnes, Loulieta Nazerai, Jan P. Christensen, Allan R. Thomsen

**Affiliations:** Department of Immunology and Microbiology, University of Copenhagen, Copenhagen, Denmark

**Keywords:** immune surveillance, CNS, tissue resident memory T cells, adenovectors, viral infection

## Abstract

While the brain is considered an immune-privileged site, the CNS may nevertheless be the focus of immune mediated inflammation in the case of infection and certain autoimmune diseases, e.g., multiple sclerosis. As in other tissues, it has been found that acute T-cell infiltration may be followed by establishment of persistent local T-cell memory. To improve our understanding regarding the regulation of putative tissue resident memory T (Trm) cells in CNS, we devised a new model system for studying the generation of Trm cells in this site. To this purpose, we exploited the fact that the CNS may be a sanctuary for adenoviral infection, and to minimize virus-induced disease, we chose replication-deficient adenoviruses for infection of the CNS. Non-replicating adenoviruses are known to be highly immunogenic, and our studies demonstrate that intracerebral inoculation causes marked local T-cell recruitment, which is followed by persistent infiltration of the CNS parenchyma by antigen specific CD8^+^ T cells. Phenotypical analysis of CNS infiltrating antigen specific CD8^+^ T cells was consistent with these cells being Trms. Regarding the long-term stability of the infiltrate, resident CD8^+^ T cells expressed high levels of the anti-apoptotic molecule Bcl-2 as well as the proliferation marker Ki-67 suggesting that the population is maintained through steady homeostatic proliferation. Functionally, memory CD8^+^ T cells from CNS matched peripheral memory cells with regard to capacity for *ex vivo* cytotoxicity and cytokine production. Most importantly, our experiments revealed a key role for local antigen encounter in the establishment of sustained CD8^+^ T-cell memory in the brain. Inflammation in the absence of cognate antigen only led to limited and transient infiltration by antigen specific CD8^+^ T cells. Together these results indicate that memory CD8^+^ T cells residing in the CNS predominantly mirror previous local infections and immune responses to local autoantigens.

## Introduction

It is well-known that T cells play a key role in the pathogenesis of many infections, autoimmunity and cancer. From a developmental point of view T cells are divided into naïve, effector, and memory cells, where memory T cells are antigen primed cells that play an important role in the accelerated immune response against previously encountered antigens. Classically, memory T cells have been divided into central memory cells that recirculate between the secondary lymphoid organs and express the lymph node homing receptors CCR7 and CD62L, and effector memory cells, the function of which is to patrol the peripheral organs and rapidly infiltrate inflamed areas (1). However, recently a third and very large subset of memory T cells has been detected (2–4). These have been named tissue resident memory T cells (Trms) and represent sessile cells often autonomously maintained in close proximity to the classical ports of pathogen entry, e.g., skin and mucosal surface in the gut and lungs, where they serve as an important first line of defense against pathogens associated with these organ sites (2, 5). Trms develop from antigen primed cells that enter the relevant tissues during the effector phase and locally receive the environmental cues required to adopt the unique signature particular to these cells (5–7). Thus, Trms are characterized by their own set of surface molecules such as CD69 and CD103, both of which are assumed to play an important role in the local maintenance of the Trm population (6). CD69 antagonizes the activity of the sphingosine 1-phospate receptor (S1P1), which is a critical regulator of T cell egress from most tissues (8), and CD103, which pairs with integrin β7 and mediates binding to E-cadherin, may be involved in the positioning and retention of the cells (9), although CD103^−^ Trms have been described in several tissues including the intestines, secondary lymphoid organs and liver (10–14). Indeed, no single cell surface marker represents a definitive marker for Trms, and the ultimate approach to evaluate the degree of tissue residency of a memory population remains a comparative analysis of immune cell distribution between animals of parabiotic pairs (4). Notably, increasing evidence has shown that Trms can also be found in non-barrier tissues including the CNS (14), and may contribute critically in autoimmunity and tumor immunity (15, 16) in addition to their role in the defense against pathogens (5, 6, 17). Given that CNS represents a vulnerable organ where infections as well as tumors need to be rapidly and efficiently controlled, yet without too much collateral tissue destructive damage, this study was undertaken to establish a new model for studying the characteristics and regulation of the Trm response in the brain.

Classically, the interior of the brain has been considered an immunologically privileged site, and it is true that until antigen (e.g., in the form of a viral infection) reaches the circulatory system and in this manner may reach and subsequently activate the immune system, adaptive immunity is not efficiently invoked (18–20), unless locally positioned and primed sentinel cells react to the presence of cognate antigen. This underpins the fragility of the immune surveillance of the CNS, and prior seeding of antigen primed T cells is therefore of key importance in initiating a potent inflammatory response that may rapidly control a nascent infection. On the other hand, if the recognized antigen is expressed by our own cells, the same efficient detection mechanism may trigger an autoimmune reaction with dire consequences for the well-being of the host. The latter is what is believed to occur in patients with acute demyelinating encephalomyelitis or multiple sclerosis (MS). While it is obvious that understanding how to induce the appropriate local immunity is of critical importance in context of improving vaccination regimens, it should also be pointed out that improved insight into the role of Trms in autoimmunity may have important implications for how we should treat autoimmune diseases in the future. Preventing recruitment from the circulation would be of little consequence, if autonomously maintained local populations of Trms play the most important role in the activity of a disease like MS.

Models of CNS autoimmunity are typically hampered by the fact that the pathogenic T cells are generally of CD4^+^ T-cell type, while in humans CD8^+^ T cells dominate the CNS lesions (21). This along with the fact that the antigen specific T cells are not easily analyzed quantitatively and qualitatively calls for a new approach. Therefore, to develop a model system to study CD8^+^ T cell functioning in the CNS, we exploited the fact that the CNS may be a sanctuary for adenoviruses (22). As induction of lethal disease would clearly defy the purpose of our study, we chose to make use of adenoviruses that were made incapable of replicating through modification of critical genes. Such adenovectors are nevertheless highly immunogenic and peripheral inoculation is known to induce potent CD8^+^ T cell responses (23, 24). In our experimental set-up the immunogenicity was in some cases even further improved by tethering of the antigen to the murine invariant (Ii) chain, which increases surface antigen presentation through mechanisms not fully understood (25, 26). Using these vectors we could not only mimic a non-lethal viral infection of the CNS; as antigen expression from adenovectors is known to be relatively long-standing, the induced response could also serve as a model for an autoimmune response against the tissue/cells supporting persistence of the vector. In this study we show that intracerebral (i.c.) inoculation of non-replicating adenovectors results in efficient T-cell priming as well as potent recruitment of antigen specific CD8^+^ T cells to the CNS. The recruited cells differentiate locally and eventually carry all the hallmarks of Trms; for months this population is maintained locally in a functional state resembling that of peripheral memory T cells with the same specificity. Notably, local antigen presentation is of critical importance in the recruitment of the effector cells, which develop into a persistent local memory population, and this was found to be the case whether we looked at adenovector induced responses or those following infection with live virus.

## Materials and Methods

### Mice

Female C57BL/6 (B6, H-2^b^) wild-type (WT) mice and MHC class II knock out (KO) mice on B6 background were obtained from Taconic Farms (Ry, Denmark). IFN-γ and IFN-γ receptor (IFN-γR) KO mice were the progeny of breeder pairs originally obtained from the Jackson Laboratory (Bar Harbor, Maine, USA).

All mice used in this study were 6–12 weeks old when experiments were started and they were housed in individually ventilated cages under specific pathogen free conditions at the ALAAC accredited animal facility at the Panum Institute (Copenhagen, Denmark). Mice coming from outside sources were allowed to rest for at least 1 week before entering an experiment.

### Recombinant Adenoviral Vectors and Vaccination

E1-deleted E3 inactivated, human serotype 5 recombinant adenoviral (Ad5) vectors encoding the gene for the glycoprotein (GP) of lymphocytic choriomeningitis virus (LCMV)(AdIi-GP) or ovalbumin (OVA)(AdIi-Ova) tethered to the murine MHC class II-associated invariant chain (Ii) were used for induction of antigen-specific T cell responses as was Ad5 vectors expressing either the nucleoprotein (NP) or the RNA catalytic subunit PB1 of influenza virus [Ad-NP(inf) and Ad-PB1, respectively]. The construction of these vectors has been described previously (25, 27). An Ad5 vector encoding the gene for murine IFN-γ was used to initiate non-specific inflammation; this vector has previously been described in Kolls et al. (28) and was kindly provided to us by Trevor Owns (SDU, Denmark) (29). Adenoviral vectors were produced and quantified as previously described (25). The appropriate vaccine solutions were prepared by diluting the adenoviral vaccine stocks in phosphate-buffered saline (PBS). Prior to vaccination mice were deeply sedated with isoflourane and mice were injected intracranially (i.c.) and/or subcutaneously (s.c) in the right footpad with 30 μL containing 2 × 10^7^ pfu of the adenoviral constructs.

### Infection With Live Virus (LCMV)

LCMV Armstrong 53B (LCMV Arm) was diluted in PBS to a concentration of 10^4^ pfu/300 μL and administered intravenously (i.v.) for systemic infection; mice infected in this manner raise a very potent T-cell response dominated by CD8^+^ T cells directed against the GP and NP of LCMV and rapidly recover from the infection (30, 31). In some experiments previously infected mice were challenged i.c. using a dose of 10^3^ pfu in 30 μL; this invariably causes lethal disease in naïve mice, but previously i.v. infected mice survive the infection. Viral stock as well as organ virus levels were determined in a focus-forming assay as previously described (32).

### Single Cell Preparation

Prior to the procedure of aseptically removing the brain and spleen, mice were deeply anesthetized by intraperitoneal (i.p) injection of avertin (2,2,2 tribromoethanol in 2-methyl-2-butanol, 250 mg/kg). Spleens were removed and transferred to Hanks Balanced Salt Solution (HBSS). Single cell suspension was prepared by mincing the spleens through a 70-μm nylon cell strainer followed by centrifugation and wash in HBSS twice.

Brains were aseptically removed after intracardial perfusion with 20 mL PBS. Brains were transferred to 13 mL RPMI 1640 [containing 1% L-glutamin, 1% penicillin, 1% streptomycin, 1% 2-mercaptoethanol (2-ME) and 10% fetal calf serum (FBS)]. Single cell suspension was obtained by pressing the brains through a 70-μm nylon cell strainer. The single cell suspension was subsequently centrifuged at 400 g for 10 min at 4°C. The remaining pellet was vortexed and lymphocytes were separated on a 37% Percoll gradient during 20 min centrifugation at 2,800 g, 20°C. After aspiration of the supernatant, the remaining pellet was vortexed and washed twice in RPMI 1640 followed by one additional filtration through a 70-μm cell strainer. Finally the cells were resuspended in FACS medium (PBS, 1% BSA, 0.1% N_a_N_3_).

### Isolation of Cerebrospinal Fluid (CSF)

The mice were deeply anesthetized and exsanguinated from the axilla. Then the skin was lifted from the head, and pulled forward until the eyes were visible. Following gentle removal of the overlying muscles, the dura mater of the cisterna magna appeared at the base of the skull as a clear triangle and through here, there was access to the fourth ventricle and the CSF. By making a small slit in the dura mater, the CSF could be collected using a capillary tube that was gently placed against the hole. In a healthy mouse, up to 10 μL of CSF may be collected, while lesser volumes of CSF can be obtained from clinically affected mice (33). Any CSF visibly contaminated by blood was discarded. A Bürker-Türk counting chamber was used to enumerate the cells in the collected CSF.

### Flow Cytometric Analysis

Following preparation of single cell suspensions, approximately 2–5 × 10^6^ cells from the brain samples or 2 × 10^6^ spleenocytes were transferred to wells of U-buttomed 96-well microtiter plates. For surface staining, the cells were incubated for 20 min (4°C, in the dark) with 50 μl FACS medium (PBS, 1% BSA, 0.1% N_a_N_3_) containing 10 μL relevant fluorochome labeled tetramers (30, 31); these were kindly provided by S Buus and A Stryhn (this institute) (34). Additionally the cells were incubated for 20 min (4°C, in the dark) with 50 μL brilliant violet staining buffer (Biolegend) containing conjugated antibodies (1:100) for the relevant cell-surface markers and unconjugated CD16/32 to prevent unspecific binding. For many of the phenotypic analyses, the pattern for two different antigen specific subsets was evaluated; only subtle differences were noted. Cells to be stained for intracellular Granzyme B, Bcl-2 or Ki-67 expression were permeabilized and stained according to the FoxP3 staining protocol from BD Biosciences. After incubation, the cells were washed twice in wash media (PBS with 0.1% N_a_N_3_), resuspended in PBS and stored at 4°C until flow cytometric analysis later the same day.

### Intracellular Cytokine Staining

For evaluation of the intracellular cytokine production, cells were incubated for 5 h (37°C, 5% CO_2_) in 200 μL cell culture medium containing 50 IU/mL of IL-2 and 3 μM monensin in the presence of 1 μg/mL of the relevant peptides, LCMV GP_33−41_ or GP_276−286_ (30, 31). Control samples did not receive any peptide or were incubated in the presence of the irrelevant peptide flu NP_366_ (27). Following incubation, the cells were centrifuged (2,000 rpm, 3 min), washed with FACS buffer with monensin (PBS containing 1% BSA, 0.1% NaN3 and 3 μM monensin) and incubated for 20 min (4°C, dark) with 50 μL FACS/Monensin medium containing the relevant surface antibodies (final dilution 1:100). Cells were then washed twice with PBS/monensin medium (3 μM monensin in PBS) and fixated in 200 μL 2% paraformaldehyde (PFA) for 15 min (4°C, dark). Subsequently, cells were washed with FACS/monensin medium and incubated for 10 min (20°C, dark) with 200 μL Saponin medium (PBS containing 0,5% Saponin). Next, the cells were incubated for 20 min (4°C, dark) with 50 μL Saponin medium containing the relevant intracellular antibodies (1:100). The cells were subsequently washed twice with Saponin medium and finally resuspended in PBS and stored at 4°C until flow cytrometry analysis the same day.

Cell samples were analyzed using a FACS LSR II—or Fortessa cytometer (BD Biosciences) and the data were analyzed using FlowJo software version 10 (TreeStar); see Supplementary Figure 1 for gating strategy.

### Antibodies

The following fluorochrome-conjugated monoclonal Abs were used for flow cytometry. Surface staining: α-CD8, α-CD11b, α-CD45.2, α-CD44, α-CD103, α-CD69, α-CD49a, α-CD127, α-KLRG1, α-CD4, and α-CD107a. For intracellular cytokine staining we used: α-IFN-γ, α-IL-2, and α-TNF. Staining for intracellular molecular markers involved: α-Granzyme B, α-Bcl-2, and α-Ki-67. All antibodies were purchased from Biolegend, eBioscience or BD Bioscience as anti-mouse antibodies.

### Statistical Evaluation

GraphPad Prism software (version 7) was used for the statistical analyses. Quantitative results were compared using nonparametric Mann-Whitney *U*-test and a *p*-value of < 0.05 was considered evidence of statistically significant difference and visualized by a ^*^.

## Results

### Induction of Antigen Specific T-cell Infiltration in the CNS by Priming With Non-replicating Adenovectors

**Figure 1:**
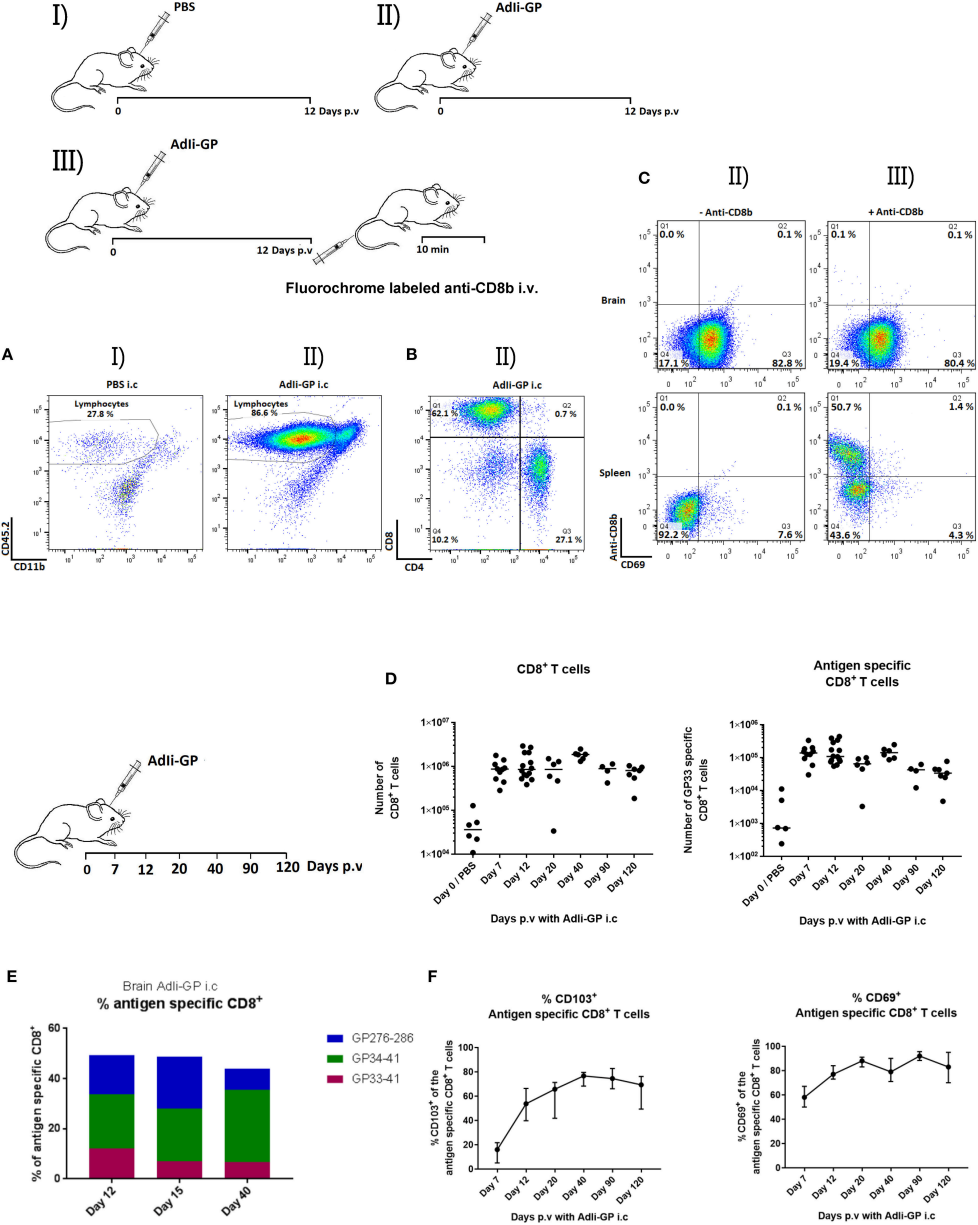
Induction of T-cell infiltration in the CNS as a result of i.c. injection with non-replicating adenovectors. Groups of mice were injected i.c. with PBS (I) or AdIi-GP (II). **(A–C)** Twelve days later, cells were harvested from the CNS and analyzed for cell surface markers by flow cytometry. Dot plots showing the gating of lymphoid cells in representative mice of groups I and II. **(A)** Representative dot plots depicting the subdivision of lymphocytes in group II mice with regard to T-cell subset markers **(B)**. **(C)** Mice were injected i.c. with AdIi-GP, and shortly before cell harvesting half the mice were injected i.v. with flourochrome labeled anti-CD8b (III), the other half served as controls (II). Following *ex vivo* staining for cell surface markers, the harvested cells were analyzed by flow cytometry; representative dot plots of gated CD8^+^ T cells from brain and spleen from 4 to 5 mice per group in at least two independent experiments are depicted **(C)**. **(D,F)** On the indicated days cells from CNS were harvested, and total numbers of infiltrating CD8^+^ T cells as well as antigen specific CD8^+^ T cells in CNS were quantified **(D)**. Percent of antigen specific cells out of total infiltrating CD8^+^ T cells measured using tetramers matching the 3 most dominant GP epitopes; medians of at least 5 mice are shown **(E)**. Expression of CD69 or CD103 on infiltrating antigen specific CD8^+^ T cells as a function of time after i.c. inoculation; group medians and ranges of groups of at least 5 mice is depicted **(F)**.

To determine if it was possible to induce a lymphocyte infiltrate in the brain by inoculation of non-replicating adenovirus, WT mice (B6) were inoculated i.c. with 2 × 10^7^ pfu of an adenovector expressing the GP of LCMV tethered to the murine Ii chain for improved immunogenicity (AdIi-GP). Controls were inoculated with PBS to control for non-specific background infiltration of the brain induced by the mechanical trauma. Brains were extracted on day 12 p.v, which is known to represent the peak in the effector response induced with this vector (25). As seen in Figure 1A, AdIi-GP inoculation resulted in a robust cell infiltrate in the brain of which about 80% appeared to represent lymphocytes; for comparison in the CNS of PBS injected mice, lymphocytes made up only 27% of the recovered cells. When we analyzed the composition of the recruited lymphocytes, we found a clear dominance of CD8^+^ T cells, thus CD8^+^ and CD4^+^ T cells were found to account for about 60 and 30%, respectively, of the cells inside the lymphocyte gate (Figure 1B). Many of the recruited T lymphocytes were recently activated as indicated by an increased expression of CD25 relative to matched cells from the spleen (data not shown).

In order to ascertain that the extracted cells were bona fide tissue infiltrating CD8^+^ T cells located in the brain parenchyma, and not marginated intravascular CD8^+^ T cells, mice were vaccinated i.c. with 2 × 10^7^ pfu AdIi-GP, and at day 12 p.v, half the mice were administered fluorochome labeled anti-CD8b i.v. 10 min prior to brain and spleen extraction (11); the recovered cells from both groups were subsequently analyzed by flow cytometry. Less than 0.2 percent of the cells harvested from the brain were labeled by anti-CD8b injection. In contrast, in the spleens of the same mice two distinct populations differing in labeling status were noted (Figure 1C): labeled cells representing cells from the red pulp and unlabeled cells representing white pulp lymphocytes (11). Together these findings validated the *in vivo* labeling protocol and verified that CD8^+^ T cells harvested from the brain were tissue infiltrating CD8^+^ T cells.

Previous studies of Trms in the lungs induced by application of similar adenovectors have revealed that local infection combined with additional peripheral priming resulted in increased recruitment to the infected lungs (27). For that reason, we wanted to investigate if this was also true for the recruitment to the brain. Consequently, four groups of mice were vaccinated either with AdIi-GP i.c., in the f.p., combined i.c. and f.p. or with PBS i.c. as a control. At day 12 p.v. brains were harvested and the cellular infiltrate was analyzed by flow cytometry. We found that systemic immunization alone, as induced by the peripheral priming (AdIi-GP f.p.), did not result in demonstrable recruitment of antigen specific CD8^+^ T cells beyond the background in PBS inoculated mice (Supplementary Figure 2). In contrast, when antigen was presented locally (AdIi-GP i.c. and AdIi-GP i.c. + f.p.) a robust CD8^+^ as well as antigen specific CD8^+^ T cell recruitment was observed. However, additional peripheral priming (f.p.) did not result in a significantly increased recruitment of antigen specific cells to the CNS compared to AdIi-GP i.c. only vaccination.

### I.c. Inoculation of AdIi-GP Induce Persistent CD8^+^ T-cell Memory in the CNS

Next, we wanted to study the longevity of the cellular infiltrate and phenotype of the recruited CD8^+^ T cells. To this end, C57BL/6 mice were inoculated i.c. with 2 × 10^7^ pfu AdIi-GP and brains were extracted at day 7, 12, 20, 40, 90, and 120 p.v and analyzed by flow cytometry. We found the recruited population of CD8^+^ T cells to be maintained until at least day 120 p.v., and analysis of their specificity revealed the presence of a permanent subset of antigen (H-2D^b^ restricted GP_33−41_) specific CD8^+^ T cells in CNS for the entire observation period (Figure 1D). To evaluate the composition of the recruited cells, we studied the total pool of antigen specific cells by additional analysis of H-2D^b^ restricted GP_276−286_-and H-2K^b^ restricted GP_34−41_ tetramer positive CD8^+^ T cells at day 12, 20 and 40 p.v. We found the total number of these antigen specific cells to account for 45–50% of the maintained population, with the percentage of GP_33−41_ specific cells slightly decreasing over time until day 40 p.v (Figure 1E). It should be added at this point that we also studied the impact of additional peripheral priming on the longevity and stability of the CNS infiltrate and failed to observe any significant impact (data not shown).

Tissue-resident T cells are often characterized by expression of the surface markers CD69 and CD103 (4). Therefore, to gain further insight into the nature of these brain infiltrating, antigen specific cells, we investigated the expression of CD69 and CD103 at the previously described time points. As expected, the percentage of antigen specific cells expressing CD69 was high from the beginning, while the percentage of antigen specific cells expressing CD103 was found to increase during the observation period, reaching a maximum level of expression on 2/3 or more of GP_33_ specific cells (Figure 1F). Phenotyping for other typical cell surface markers of tissue resident T cells at several time-points (see Supplementary Figure 3 for representative data) revealed that the infiltrating memory CD8^+^ T cells were CD44^high^ and the majority eventually expressed CD49a (VLA-1). Analyzing the expression of KLGR1 and CD127 over time we found that initially part of the recruited cells expressed KLGR1 and 10% co-expressed KLGR1 and CD127. However, by day 12 the majority lacked expression of KLGR1, and if anything, this phenotype became even more dominant at later time-points (Supplementary Figure 3 and data not shown).

Regarding the mechanisms underlying the long term stability of this population, recruited cells had up-regulated Bcl-2 and the expression of Ki-67 largely matched that of antigen primed (CD44^high^) CD8^+^ T cells in the spleen (Supplementary Figure 4) indicating significant homeostatic proliferation. Taken together these findings demonstrate that upon i.c. vaccination with a non-replicating adenovector we could induce the formation of a CD8^+^ T cell infiltrate in the brain. The recruited CD8^+^ T cells expressed the classical markers of Trms and were maintained in the brain long-term. Hence, we had established a model for further investigations of CD8^+^ tissue-resident memory cells in the brain.

### Local Antigen Is Required for Maximal Recruitment of Antigen Specific CD8^+^ T Cells, Up-regulation of CD103 and Persistent Memory

**Figure 2:**
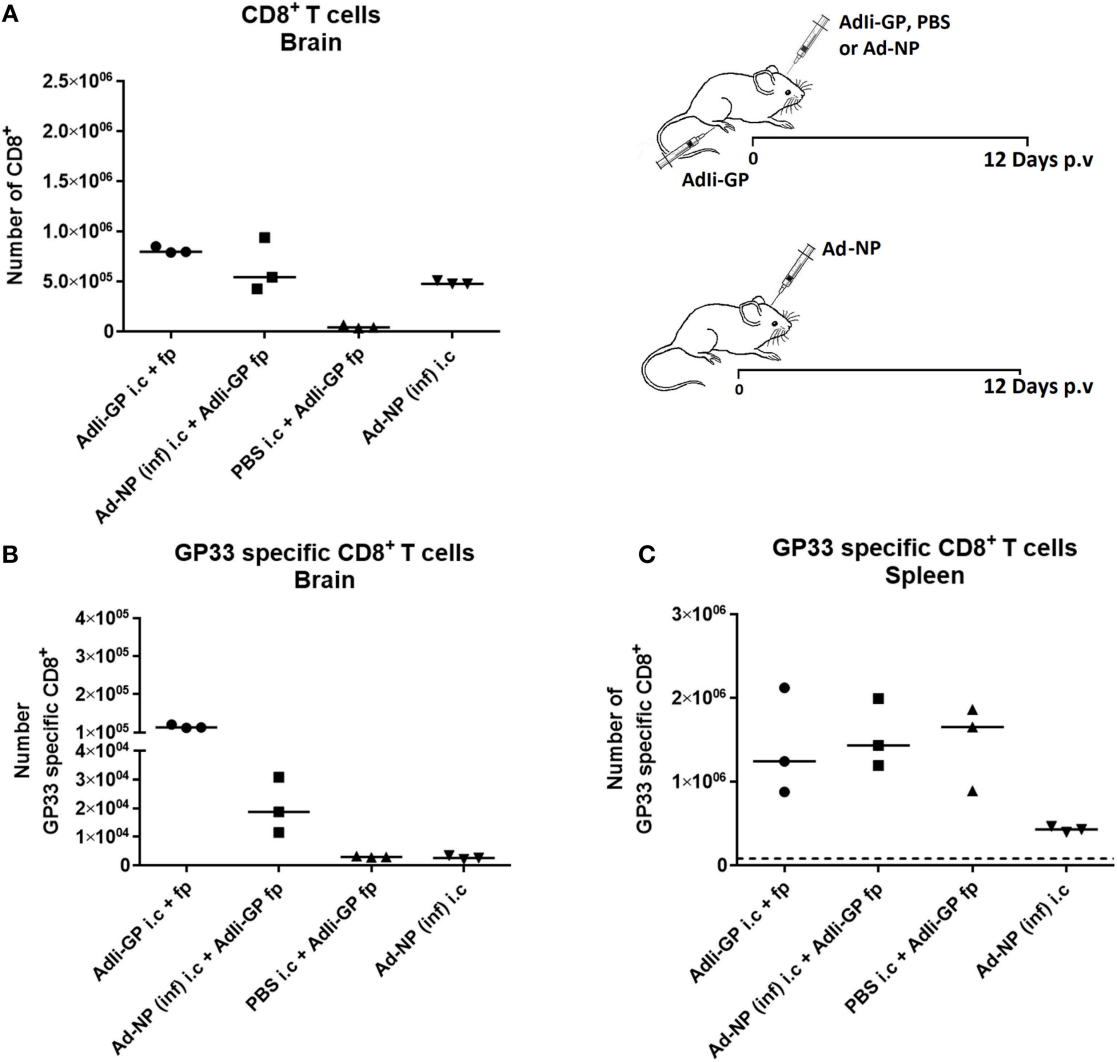
Importance of local antigen recognition for substantial accumulation of antigen specific CD8^+^ T cells in CNS. Groups of mice were injected both i.c. and f.p. or i.c. only with relevant (AdIi-GP) or irrelevant [Ad-NP(inf)] adenovectors or PBS as indicated. Twelve days later total numbers of infiltrating CD8^+^ T cells **(A)** as well as numbers of antigen specific CD8^+^ T cells in CNS **(B)** were enumerated. Numbers of matching antigen specific CD8^+^ T cells in spleen of the same animals has been included for comparison **(C)**. Symbols represent individual mice. Vertical bars represent group medians. Similar results were obtained in a second experiment using another irrelevant vector.

In some, but not all tissues the formation of a Trm population requires interaction with antigen in the local environment (35–41). Consequently, we wanted to evaluate the relative roles of inflammation and antigen recognition in the formation of a CD8^+^ T-cell infiltrate in the CNS. For that purpose C57BL/6 mice were injected with 2 × 10^7^ pfu AdIi-GP in the foot pad (f.p.). This was combined with i.c. inoculation of either the same vector or an adenovector expressing an irrelevant epitope, the NP_366_ from influenza nucleoprotein [Ad-NP(inf)]. For control, groups of mice were given only Ad-NP(inf) i.c. or PBS i.c. combined with a f.p. priming of 2 × 10^7^ pfu AdIi-GP. Brains and spleens of all the mice were harvested on day 12 p.v. and analyzed for the total number of recruited CD8^+^ T cells and for the presence of GP_33_ specific CD8^+^ T cells using tetramers. In accordance with the previous findings, i.c. inoculation of both vectors caused significant local CD8^+^ T cell recruitment to the CNS (Figure 2A). However, when analyzing the specificity of the recruited cells (Figure 2B), we found that only in the presence of the relevant (GP_33−41_) epitope locally and not merely as a result of adeno-vector induced bystander inflammation, were GP_33_ specific CD8^+^ T cells recruited to the brain. As a control for the efficiency of peripheral priming of antigen specific CD8^+^ T cells, we analyzed the presence of GP_33_ tetramer positive CD8^+^ T cells in the spleen (Figure 2C). These results verified that the observed difference in antigen specific recruitment to the brain did not reflect differences in numbers of systemically primed antigen specific T cells. Also it should be noted that Ad-NP(inf) served as a perfectly efficient signal for recruiting NP366 specific cells (Supplementary Figure 5), underscoring the importance of antigen specificity in T-cell recruitment. Using another irrelevant Ad-vector, AdIi-Ova, instead of Ad-NP(inf) for the i.c. inoculation gave matching results, thus further underpinning the importance of local cognate antigen recognition (data not shown).

**Figure 3:**
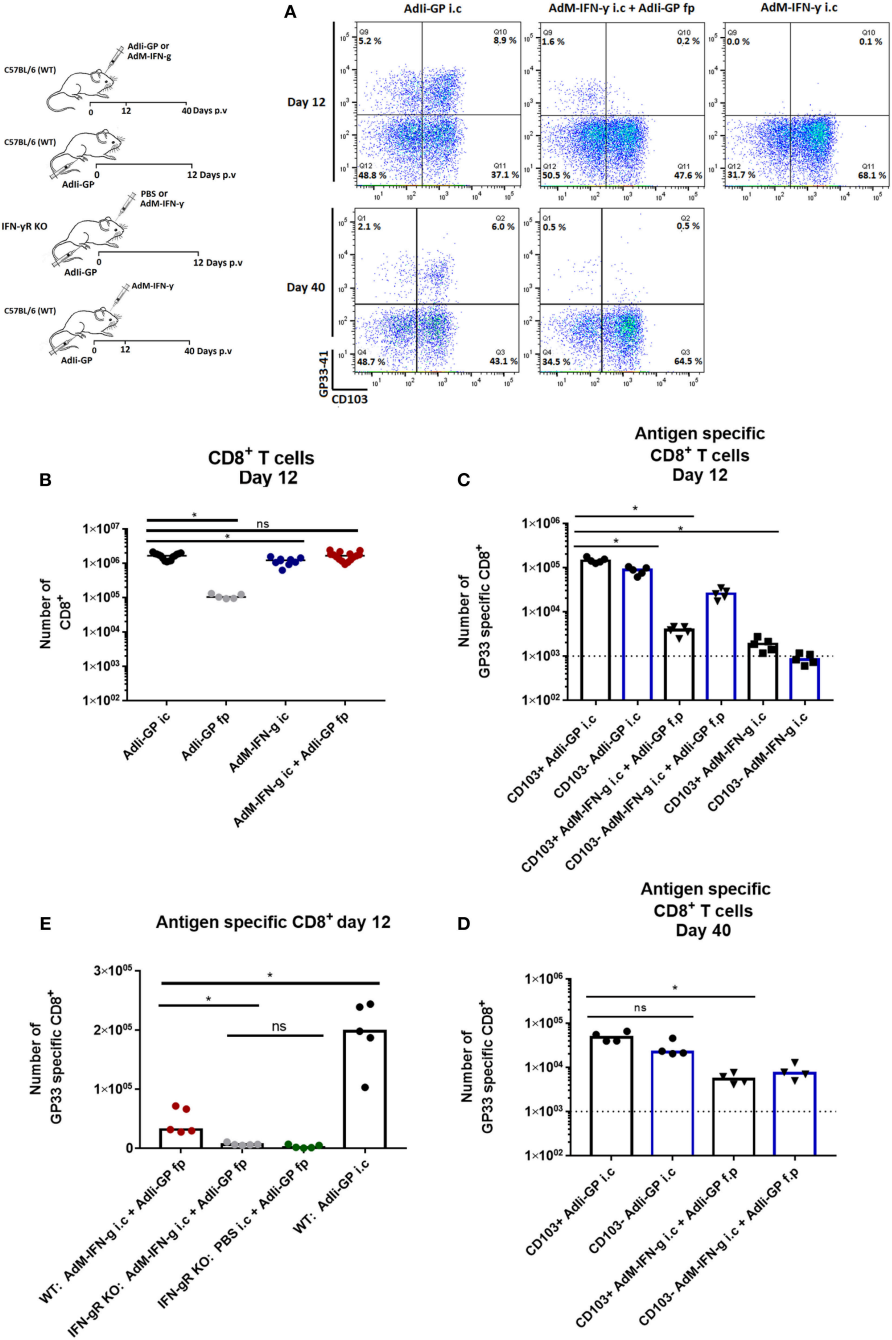
Role of local inflammation in the accumulation of antigen specific CD8^+^ T cells in CNS. Groups of wild type mice were injected i.c. and/or f.p. with adenovectors (AdIi-GP or an IFN-γ producing vector, AdM-IFNg) or PBS as indicated. Twelve and forty days later infiltrating CD8^+^ T cells were harvested. **(A)** Representative dot plots of gated CD8^+^ T cells. **(B,C)** Total numbers of infiltrating CD8^+^ T cells **(B)** as well as numbers of antigen specific CD8^+^ T cells **(C)** in the CNS on day 12 after i.c. inoculation. **(D)** Forty days after i.c. inoculation mice of the only two groups with significant antigen specific T-cell infiltration on day 12 were analyzed and antigen specific CD8^+^ were enumerated. **(E)** Groups of wild type (WT) and IFN-γ receptor deficient (IFN-γR) mice were inoculated as indicated and infiltrating antigen specific CD8^+^ T cells were enumerated 12 days later. Symbols represent individual mice. Vertical bars represent group medians. Results have been pooled from two experiments. *P<0.05.

To further explore the role of unrelated inflammation in CD8^+^ T-cell recruitment, we made use of an adenovector construct encoding the gene for murine IFN-γ causing infected cells to produce this proinflammatory cytokine (28). C57BL/6 mice were injected with 2 × 10^7^ pfu AdM-IFN-γ i.c. only (negative control) or in combination with peripheral AdIi-GP priming (f.p.). To gauge the response against that associated with local antigen expression, another group of mice were injected with AdIi-GP i.c. Additionally, mice vaccinated only with AdIi-GP in the foot pad were included as controls for background infiltration. We found that while AdM-IFN-γ did induce significant CD8^+^ T cell infiltration measurable 12 days after i.c. inoculation (Figure 3B), the frequency of peripherally primed GP33 specific cells attracted to the brain was significantly lower than in the case where antigen was present locally (Figure 3C). Moreover, it was observed that the previously noted up-regulation of CD103 on the GP33 specific CD8^+^ T cells only occurred in the context of local GP expression in the brain (Figures 3A,C). Probably associated to this, the infiltration of GP33 specific cells induced by proinflammatory cytokine was less stable over time compared to when antigen was present locally (Figures 3A,D).

To ascertain that the limited inflammation driven by the IFN-γ producing vector primarily reflected the action of the cytokine, C57BL/6 and IFN- γR KO mice were similarly vaccinated with 2 × 10^7^ pfu AdIi-GP in the f.p. combined with an i.c. vaccination with either AdM-IFN-γ or PBS. WT mice vaccinated i.c. with AdIi-GP were included as a positive control. When the cell infiltrate was compared on day 12 p.v., we found significantly fewer antigen specific CD8^+^ T cells recruited to the brain of IFN-γR KO mice (Figure 3E). Together the above findings emphasize, that while IFN-γ alone do not cause the same level of specific CD8^+^ T cell recruitment as observed in the presence of antigen, IFN-γ is, nevertheless, a potential inducer of significant T-cell recruitment.

The above finding raised the question as to which role IFN-γ play in the antigen driven cell accumulation. To test this, C57BL/6 and IFN-γR KO mice were similarly inoculated with 2 × 10^7^ pfu AdIi-GP i.c. When the magnitude of local CD8^+^ T-cell infiltration was analyzed 12 days later, we found little or no impairment regarding CNS infiltration in IFN-γR KO mice, and similar results were obtained in IFN-γ KO mice (Supplementary Figure 6). Relative to wild type mice, we did find a slight shift in the composition of antigen specific cells with regard to epitope specificity. However, this is not surprising given the importance of this cytokine in regulating immunodominance (42). In conclusion, our data show that IFN-γ is not pivotal in the context of antigen driven CD8^+^ T-cell accumulation in the brain; this is in full accordance with what we have previously observed with regard to acute virus-induced meningitis (43).

### CD4^+^ T-cell Help Is Not Required for Recruitment of Antigen Specific CD8^+^ T Cells to the CNS

Conflicting results have been obtained regarding the role of CD4^+^ T cells in the generation and maintenance of CD8^+^ Trms (39, 40, 44–47). We therefore wondered what role, if any, do CD4^+^ T cells play in the recruitment and maintenance of antigen specific CD8^+^ T cells under the conditions of our model. To examine the relevance of CD4^+^ T cells, MHC-II KO mice and C57BL/6 mice were vaccinated with 2 × 10^7^ pfu AdIi-GP i.c. and 12 and 40 days p.v. brains and spleens were extracted and analyzed for the presence of antigen specific CD8^+^ T cells. The number of antigen specific CD8^+^ T cells found in the spleen in both groups of mice was then compared with the number extracted from the brain. In this context it should be added that we have previously found that the CD8^+^ T cell response induced by our invariant chain associated antigen is virtually independent of CD4^+^ T cell help (25, 26). Thus, the impact of CD4^+^ T cells on the recruitment and local maintenance of CD8^+^ T cells could be directly evaluated.

**Figure 4:**
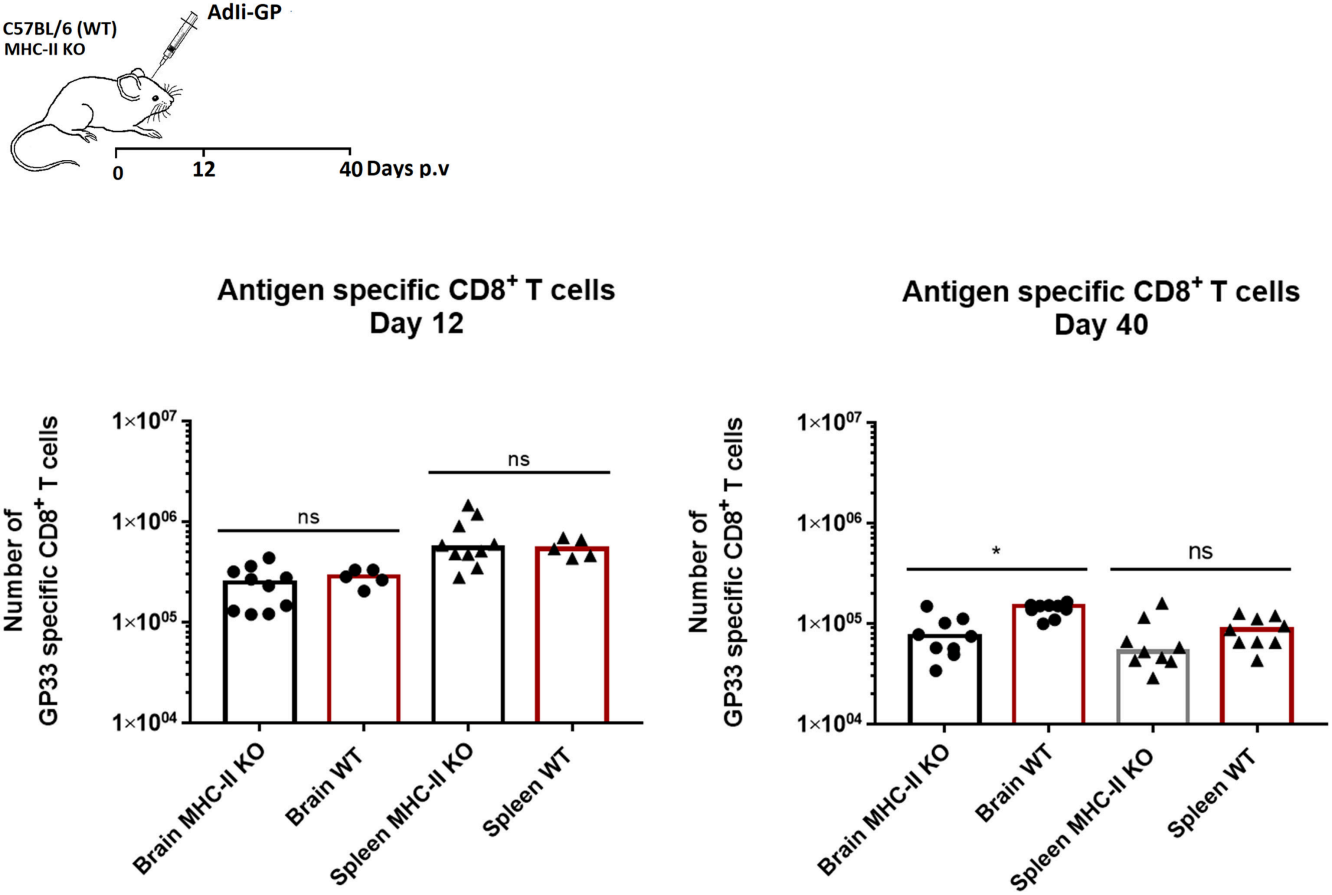
CD4^+^ T-cell help is redundant for establishment of antigen specific CD8^+^ T-cell memory in the CNS. Groups of wild type (WT) and MHC class II deficient (MHC II KO) mice were injected i.c. with adenovector. Twelve and forty days later antigen specific CD8^+^ T cells in CNS and spleen were enumerated using tetramers. Symbols represent individual mice. Columns represent group medians. Results have been pooled from two independent experiments. *P<0.05.

The results clearly showed that the initial recruitment of antigen specific CD8^+^ T cells to the brain was similar in MHC-II KO and wild type mice (Figure 4). However, on day 40 p.v., slightly fewer CD8^+^ T cells were present in the CNS of the KO mice and the same trend was seen in the periphery. Thus, help do not seem to play a pivotal role in the recruitment of adenovector induced CD8^+^ T cells, while the maintenace of this population in both spleen and CNS was slightly impaired in the absence of CD4^+^ T cells—as previously documented (26). However, consistent with earlier reports regarding lung Trms (45), we find that in CD4^+^ T cell deficient mice relatively fewer of the recruited CD8^+^ T cells express CD103, resulting in a minor shift in the balance between accumulated CD103^+^ and CD103^−^ antigen specific CD8^+^ T cells (data not shown). Please also note that in the memory phase (day 40 p.v.), more antigen specific memory T cells are recovered from the CNS than the spleen, underscoring that the Trm population contract to a lesser degree than the peripheral memory CD8^+^ T-cell population.

### Persisting Memory CD8^+^ T Cells Are Functionally Competent

**Figure 5:**
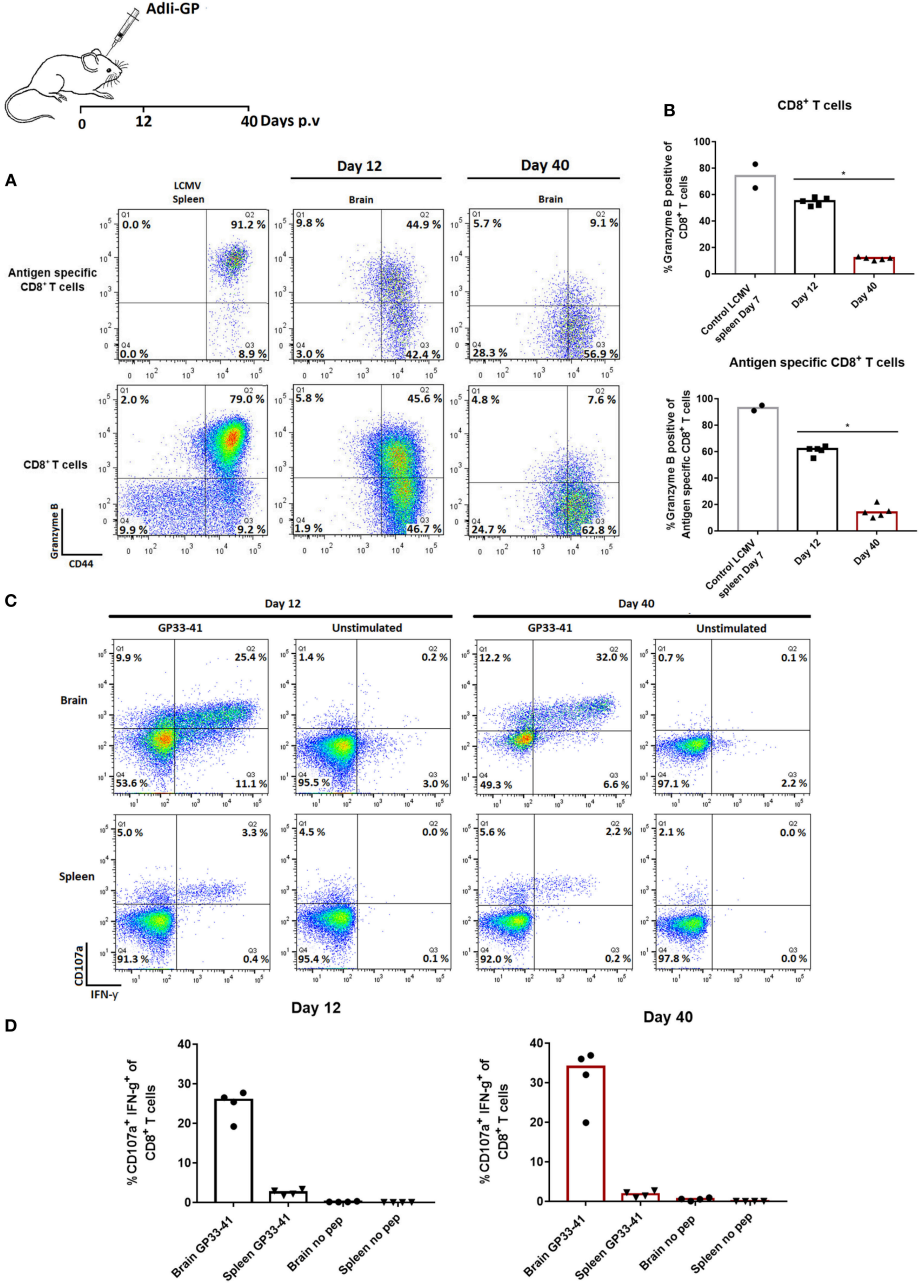
Functionality of CNS infiltrating memory CD8^+^ T cells early and late after priming. Groups of 4–5 mice were injected i.c. with AdIi-GP. Twelve and forty days later CNS infiltrating cells were harvested. **(A,B)** Total CD8^+^ T cells as well as antigen specific CD8^+^ T cells were analyzed for expression of Granzyme B. Day 7 primed CD8^+^ T cells from an LCMV infected mouse have been included to set the cut-off for expression of Granzyme B. Representative plots **(A)** and percentage of Granzyme B positive cells in individual mice **(B)**. Symbols represent individual mice. Columns represent group medians. Similar results were obtained in a second experiment. **(C,D)** Alternatively, both CNS infiltrating cells and splenocytes were harvested and stimulated *ex vivo* with relevant peptide in the presence of anti-CD107a. Following incubation, cells were surface stained, permeabilized and stained for intracellular IFN-γ. Representative plots **(C)** and percentage of degranulating (CD107a^+^) IFN-γ^+^ cells in individual mice **(D)**. Similar results were obtained in a second experiment. *P<0.05.

Tissue-resident cells found in the brain parenchyma have been found to respond to lymphocytic choriomeningetis (LCMV) challenge by perforin and Granzyme B mediated killing (47). On that note, we examined the content of Granzyme B in the cytotoxic granules of the recruited CD8^+^ T cells by intracellular staining for Granzyme B. This was done at day 12 and 40 after vaccination with 2 × 10^7^ pfu AdIi-GP i.c. As LCMV is known to elicit a very efficient cytotoxic CD8^+^ T cell response (48), splenocytes from a mouse infected with this virus 7 days earlier were used as a positive control to set the cut-off for Granzyme B positive and negative cells. The data show that at day 12 p.v. around half (45–50%) of the infiltrating (at this time mainly effector) CD8^+^ T cells were positive for this molecule, whereas the fraction of positive cells had decreased markedly (to about 15%) in the memory population day 40 p.v. (Figures 5A,B).

To get further insight into the functional capacity of the maintained population of CD8^+^ T cells, we investigated the induced surface exposure of CD107a [an indicator of the degranulation capacity (49)] as well as intracellular accumulation of IFN-γ following short term *ex vivo* peptide stimulation. Both splenic CD8^+^ T cells and CD8^+^ T cells extracted from the brain were analyzed and compared in terms of their functional capacity. Unstimulated samples were included as controls, and < 0.2% of the cells co-expressed IFN-γ and CD107a (Figures 5C,D). However, in response to *ex vivo* peptide stimulation, it was found that both in the effector and memory population from CNS, at least 25% of CD8^+^ T cells responded by co-expression of CD107a and intracellular IFN-γ (Figures 5C,D). Except for a lower frequency of reactive cells in the circulation, the response in splenocytes followed that of CNS derived cells. Thus, despite low levels of preformed Granzyme B, both degranulation and cytokine production could be activated in late resident cells. This is similar to the situation for peripheral memory cells (49), and indicates that the CD8^+^ T cells in the brain represent a fully functional CD8^+^ T-cell population both at the early effector phase and later in the memory phase.

Previous studies on the functional capacity of the CD103^+^ and CD103^−^ tissue resident CD8^+^ T cells have shown diverging results depending on the tissue of origin (7, 12, 14, 47, 50, 51). It was therefore of interest to analyze whether any functional differences exist between these two populations extracted from the brain parenchyma of adenovector inoculated mice. To do so, we analyzed the production of IL-2, TNF-α, and IFN-γ in response to short term *ex vivo* peptide (GP_33−41_ and GP_276−286_) stimulation 12 days after vaccination with 2 × 10^7^ pfu AdIi-GP i.c. While the magnitude of the response observed following stimulation with GP_276−286_ was lower compared to GP_33−41_, no significant difference in the ability of CD103^+^ and CD103^−^ CD8^+^ T cells to produce cytokines *ex vivo* was observed for either of the T-cell specificities tested (Supplementary Figure 7).

### Minimal Accumulation of Virus-Specific CD8^+^ T Cells in the CNS Following Systemic Viral Infection

In the previous experiments, using a non-lethal model for direct viral infection of the CNS we have demonstrated that local antigen presentation is essential to the formation of a potent local Trm response. However, in most cases viral infections reach the CNS following previous systemic infection. Here the situation might be different from that studied above given the extended presence of proinflammatory cytokines and a high number of antigen specific T cells in the circulation of systemically infected hosts. Therefore, we wanted to extend our studies to a situation with potential infection of the CNS subsequent to systemic infection. For that purpose we chose i.v. infection with LCMV. LCMV is a potentially neurotropic virus that causes lethal meningitis when injected i.c. whereas i.v. inoculation is considered not to result in CNS infection (33, 52). We confirmed that i.v. infection with 10^4^ PFU of LCMV Arm does not results in detectable infection of the CNS (< 200 pfu/gm organ), however, when we monitored the CSF for leukocyte infiltration, we found clear evidence of meningeal inflammation as evidenced by low, but significant leukocyte infiltration compared to uninfected controls 8 days after virus inoculation (Supplementary Figure 8). The cell accumulation was delayed compared to that associated with classical LCMV induced CNS infection following i.c. infection, and cell numbers were also much lower (peaking around 10^3^ vs. 10^4^ cells/μl, for i.v. and i.c. inoculated mice on days 8 and 6 post infection, respectively) [(33) and Fonnes et al., unpublished data].

**Figure 6:**
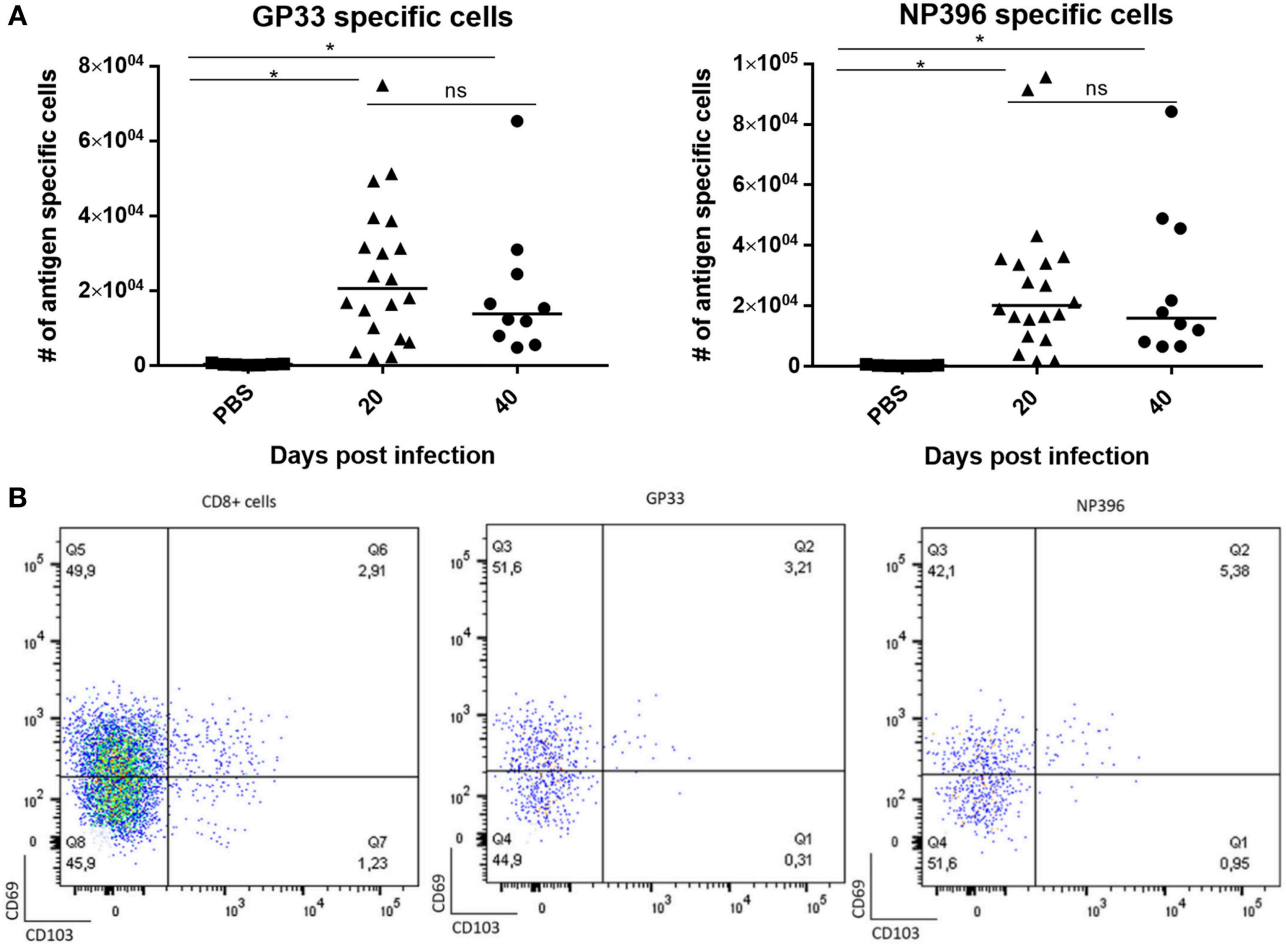
Limited CD8^+^ T-cell memory in CNS of mice with systemic viral infection. Mice were injected i.v. with 10^4^ pfu LCMV Arm or PBS and on the indicated days after inoculation, infiltrating cells from the CNS were harvested and enumerated. **(A)** Numbers of antigen specific CD8^+^ T cells targeting two major viral epitopes. Symbols represent individual mice. Vertical bars represent group medians. **(B)** Representative dot plots of total infiltrating CD8^+^ T cells as well antigen specific CD8^+^ T cells; the phenotype of gated cells are depicted with regard to expression of CD69 and CD103. Results have been pooled from two independent experiments. *P<0.05.

Based on our previous findings we hypothesized that this low grade inflammation could be a consequence of proinflammatory cytokines in the circulation, in particular IFN-γ. However, this was not the case as matching results were obtained in IFN-γ KO mice. Thus, like it may also be observed in human patients, systemic viral infection could lead to low-grade meningeal infiltration without overt local infection, raising the question if this sufficed for subsequent parenchymal infiltration eventually leading to prolonged virus specific CD8^+^ T-cell memory in the CNS. To test this i.v. infected mice were sacrificed 20 and 40 days post infection, and their brains were analyzed following the same protocol as described previously. Low, but statistically significant increased numbers of CD8^+^ T cells were recovered from the CNS (Figure 6A). Notably, when applying *in vivo* labeling with fluorochrome conjugated anti-CD8 antibody to discriminate marginated intravascular cells from infiltrating cells, we found that most of the recovered cells were not labeled *in vivo* and thus could be considered bona fide infiltrating cells (data not shown). Analysis of their specificity revealed that CD8^+^ T cells directed toward two major epitopes of LCMV together accounted for around 20–25% of the CD8^+^ T cells present. However, unlike the cells found following deliberate i.c. infection (cf. previous results), only around half the cells were CD69^+^ and very few CD103^+^ (Figure 6B). This could suggest that a large proportion of the recovered cells were not Trms, but circulating effector memory cells non-specifically infiltrating the CNS parenchyma on the basis of their high numbers in these recently infected mice. Most likely these cells did not receive the local cues needed for Trm differentiation, particularly antigen interaction (cf. our earlier findings).

### Transient Local Antigen Expression Suffices for Establishment of Persistent CD8^+^ T-cell Memory in the CNS

These observations presented provided a strong case for the importance of local antigen presentation also following infection with live virus. To test this further, we modified our LCMV model, since only mice immunized beforehand can survive an i.c. infection with LCMV and thus generate local memory CD8^+^ T cells in the brain. Therefore, we proceeded by infecting mice i.v. followed by an otherwise lethal i.c. infection (10^3^ pfu) either 7 or 30 days later. In these cases the accelerated immune response prevented a lethal outcome. CNS infiltration was analyzed 3 weeks later when the mice had fully recovered from the clinical disease nevertheless associated with the i.c. challenge.

**Figure 7:**
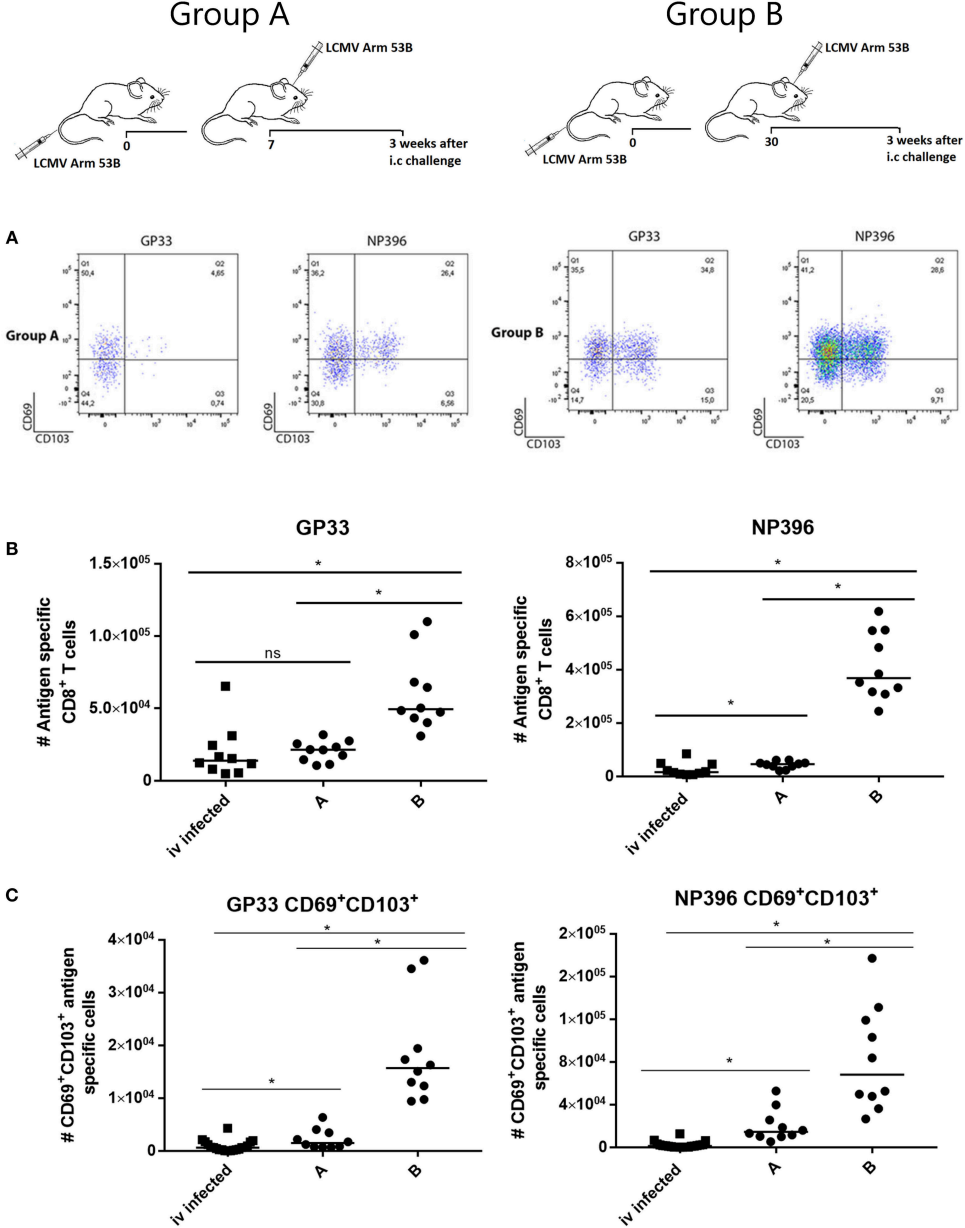
Local re-challenge induces accumulation of antigen specific CD8^+^ T cells in CNS. Mice were primed by i.v. injection of 10^4^ pfu LCMV Arm and challenged i.c. 7 (group A) or 30 (group B) days later with 10^3^ pfu of the same virus. Three weeks after i.c. challenge (the clinical impact of the challenge is depicted in (Supplementary Figure 7), CNS infiltrating cells were harvested and analyzed. **(A)** Representative dot plots of gated antigen specific CD8^+^ T cells. **(B)** Numbers of antigen specific CD8^+^ T cells recovered from CNS. **(C)** Numbers of CD69^+^CD103^+^ antigen specific CD8^+^ T cells recovered from CNS. Mice infected only i.v. have been included for comparison, these are the same as in Figure 6. Symbols represent individual mice. Vertical bars represent group medians. Results have been pooled from two independent experiments. *P<0.05.

Following i.c. rechallenge, we found more virus-specific CD8^+^ T cells in the CNS, particularly NP396 specific cells—as expected for a secondary LCMV response [(31) and own unpublished observation]—and in the group (group B) with the most severe clinical course (cf. Supplementary Figure 9). Perhaps more important, the increased infiltration was associated with increased generation of bona fide (CD69^+^CD103^+^) Trms in the CNS (Figure 7). Again the extracted cells were not labeled by i.v. injection of fluorochome labeled anti-CD8b (data not shown). Similar results were obtained in mice analyzed 7 weeks after i.c. challenge (Supplementary Figure 10). Since LCMV challenge in contrast to adenovector inoculation causes only transient antigen presentation, these results indicate that local antigen interaction is required for establishment, but not maintenance of local T cell memory in the CNS.

To exclude the possibility that the formation of Trms in CNS was merely a reflection of immunological restimulation and not presentation of viral antigen in CNS, some mice were reinfected i.p. instead of i.c. In this case we did not find any increase in CNS infiltrating cells relative to background (Supplementary Figure 11).

**Figure 8:**
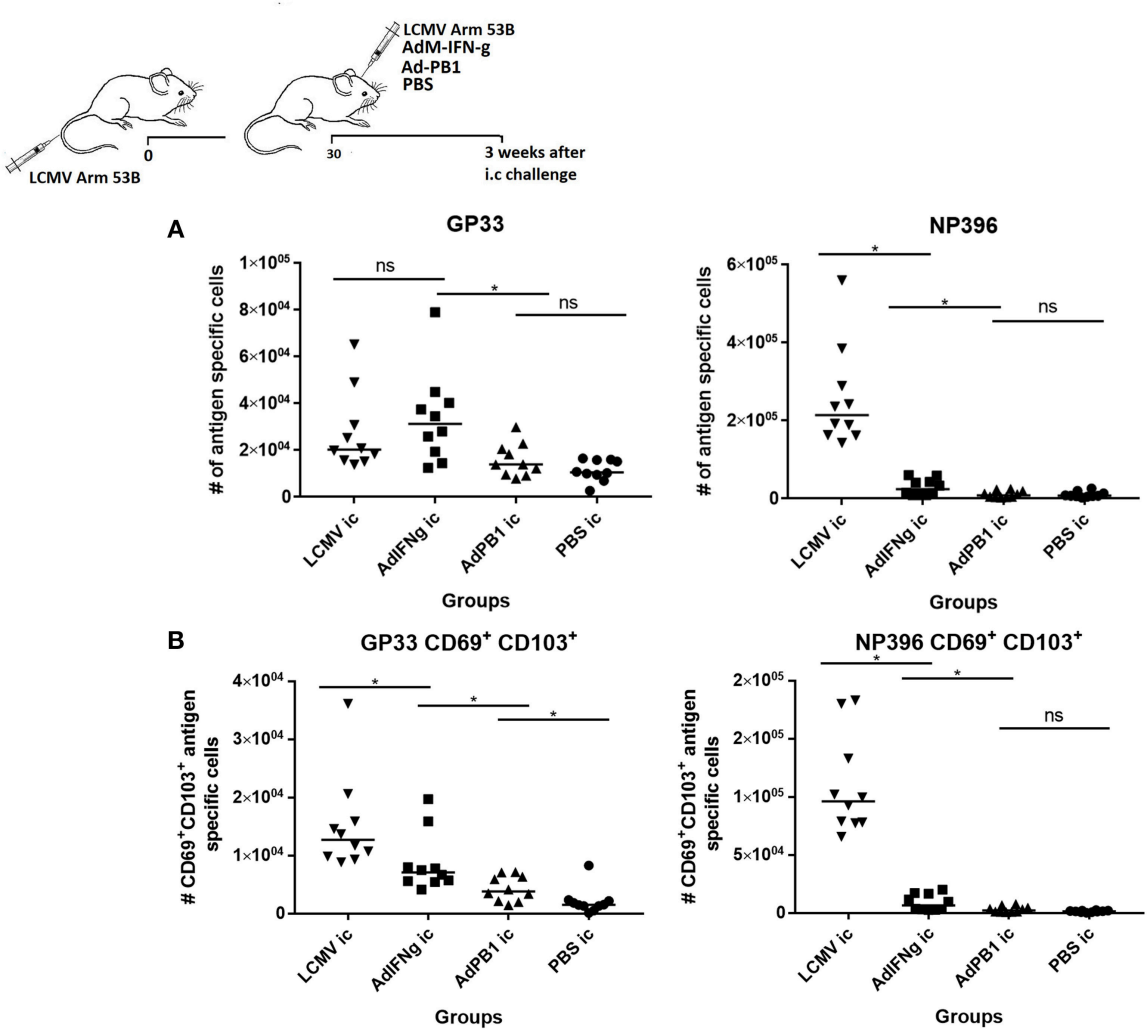
Inflammation without local antigen encounter induces limited accumulation of antigen specific CD8^+^ T cells in CNS. Mice were primed by i.v. injection of 10^4^ pfu LCMV Arm. Thirty days later mice were inoculated i.c. as indicated. After further three weeks, remaining CNS infiltrating cells were harvested and total antigen specific **(A)** as well as antigen specific CD69^+^CD103^+^ CD8^+^ T cells **(B)** were enumerated. Symbols represent individual mice. Vertical bars represent group medians. Results have been pooled from two independent experiments. *P<0.05.

Finally, to evaluate seperately the importance of local inflammatory cues in establishing persisting CD8^+^ T-cell memory in the CNS following a systemic viral infection, mice were infected with LCMV i.v. and 30 days later injected i.c. with either AdM-IFN-γ, an adenovector encoding an unrelated antigen or PBS; as positive controls immunized mice were challenged with 10^3^ pfu LCMV i.c. as above. Three weeks after the i.c. inoculation the CNS was analyzed for presence of infiltrating cells. While AdM-IFN-γ did cause some CNS infiltration of antigen specific CD8^+^ T cells (Figure 8A), none of the adenovectors induced a substantial population of LCMV specific CD69^+^CD103^+^ Trms. For comparison, mice infected i.c. with LCMV had high numbers of these cells in the CNS (Figure 8B— as previously observed cf. Figure 7).

## Discussion

In this study we have described and employed a new model system for studies of T-cell mediated immune surveillance of CNS. Here we find that i.c. inoculation of non-replicating adenovectors may be used to induce a substantial CNS infiltration dominated by CD8^+^ T cells. Although the question of the origin of the recruited antigen specific cells was not directly addressed in this study, previous studies on viral infections of the CNS have documented that these cells most likely were first activated in the secondary lymphoid organs such as the deep cervical lymph nodes and spleen as a result of overflow of the virus inoculum (volume 30 μL) into the circulation (53–56). Following local differentiation, large numbers of the generated antigen specific CD8^+^ T cells were then released into the circulation and from here attracted to the original site of virus inoculation. Most of the recruited cells were CD44^+^ and rapidly became CD69^+^, while CD103 was only expressed on few of the early infiltrating CD8^+^ T cells. However, with time—either as a result of preferential retention or local differentiation-, an increasing number of infiltrating antigen specific CD8^+^ T cells co-expressed CD69 and CD103. Even though neither of these markers are absolutely needed to be expressed on Trm cells (12, 57), a significant subset do so in most organs (4), including the CNS (14, 47). A large proportion of the infiltrating memory cells also expressed CD49a, another molecule often found on Trms. Already at day 7 after i.c. inoculation a large proportion of the recruited cells typed as memory precursor effector cells (MPEC) as determined by expression of CD127, but not KLRG-1.Whether the minority population of KLGR1^+^ cells present at this time die, migrate out or down-regulate the expression of KLGR1, we cannot say, but already by day 12 (data not shown) virtually no local CD8^+^ T cells express this marker. Development from KLRG1^−^ precursors has previously been described for skin Trms (58). Nevertheless, more CD8^+^ T cells from CNS than in the spleen expressed moderately reduced levels of CD127 compared to the CD127^high^ population in either place; what this implies is not clear at present. However, it might be relevant to note that Trms persisting in the brains of cytomegalovirus infected newborn mice have low expression of CD127 (51). Perhaps chronic antigen expression is the common denominator driving the reduction in CD127 expression in both cases. Similarly, the prolonged increase in expression of Ki-67 could be a reflection of antigen persistence as we have observed the same in the lungs of adenoimmunized as compared to flu immunized animals (Uddback et al., manuscript in prep). Regarding Bcl-2, we noted an increased expression with time of this anti-apoptotic molecule as expected following transition into a stable memory population (59). Functionally, about half of the earliest recruited cells expressed high levels of Granzyme B. The expression of this molecule decreased with time, but the cells retained the capacity to rapidly respond to cognate peptide with degranulation and production of cytokines, in particular IFN-γ, but subsets also produced TNF-α and IL-2. Thus, unlike some reports suggesting that Trms from the brain show functional deficits after dissociation from the tissue (14), our results indicate that persisting cells retain the normal functional capabilities described for memory CD8^+^ T cells *ex vivo* (49). In accordance with this view, the analysis of CNS derived memory CD8^+^ T cells completely matched that of memory CD8^+^ T cells harvested from spleen.

CD8^+^ Trm populations differing in expression of CD103 have been reported to vary in functionality. However, based on analysis of the capacity for cytokine production we did not observe any differences, nor did we find any difference in Bcl-2 expression (data not shown), which is unlike what has been reported by Wakim et al. in their study on VSV induced encephalitis (14). The proportion of CD103^+^ antigen specific CD8^+^ T cells increased between day 7 and 20 post inoculation and then stabilized around 75%. Given that adenovectors tend to persist for several months at the site of inoculation (60–62), this is an interesting observation, since some studies have indicated that persistent antigen impedes CD103 up-regulation (35, 63). Notably, we saw little or no up-regulation of CD103 on antigen specific cells recruited in the absence of cognate antigen. Overall, these observations are consistent with the contention that local triggering of the recruited T cells through their TCR, directly or indirectly, plays an important role in regulating the expression of this adhesion molecule (14).

A main finding of this study is that the recruitment of maximal numbers of antigen specific CD8^+^ T cells to the CNS critically depends on transient encounter with cognate antigen *in situ*, as also suggested by others (14, 41). However, unlike recent results from cytomegalovirus infected mice (51), our data reveal that productive infection is not essential. Following antigen recognition part of the infiltrating antigen specific effector cells differentiate locally into CD69^+^CD103^+^ double positive cells, which are stably maintained locally for several months. It has been found that even naïve cells may enter a number of uninfected/uninflamed tissues (64), and strong systemic infection, lymphopenia-induced proliferation or repeated immunization seem to drive a broad anatomical dissemination of T cells (4, 39, 65). However, regarding the CNS our results show that the presence of high numbers of activated CD8^+^ T cells in the circulation, as found in LCMV infected mice (31), do not suffice for substantial infiltration by these cells. Similar to the situation in the lungs (38), the induction of unrelated local inflammation, whether by inoculation of an IFN-γ producing adenovector or by cognate recognition of unrelated antigen by neighboring T cells did result in some local cell recruitment, but the accumulation of antigen specific CD8^+^ T cells was substantially lower and more transient than in the case of local cognate antigen recognition. It is not clear from our results whether this reduced infiltration primarily reflects a defect in actual recruitment of cells or less efficient retention and/or expansion of cells not recognizing local antigen. However, the observation that some antigen specific T cells are nevertheless attracted by IFN-γ, but fail to up-regulate expression of the adhesion molecule CD103 and that this is associated with less stable antigen specific T-cell infiltration, could be taken to support the interpretation that impaired local cell retention is a key factor. The implications of this observation is that local triggering of the recruited T cells through their TCR, directly or indirectly, play an important role in the formation of a persistent antigen specific CD8^+^ T-cell memory population in the CNS. That this is not simply an artifact of our adenovector model system is indicated by the fact that even a highly immune activating systemic viral infection like LCMV is associated with limited infiltration by antigen specific CD8^+^ T cells, most of which—based on phenotypic analysis—do not transform into bona fide Trms unless followed by direct CNS challenge. Even the induction of local non-specific inflammation in the systemically infected mice (by use of an IFN-γ producing adenovector or unrelated antigen interaction) did not lead to formation of a stable antigen specific memory T-cell population in the CNS. In further support of a unique role for local antigen recognition in the CNS, it should be mentioned that we have ourselves made similar observations in the context of systemic Zika virus infection (Nazarai et al., unpublished observation), and studies on infection with neurotropic coronavirus have pointed to the same conclusion (66). Indeed, the importance of local antigen has also been documented in other tissues, e.g., skin (40). Notably, even though cognate antigen recognition is pivotal for establishment of substantial local memory in CNS, antigen seems to be redundant in order to sustain T-cell memory, as indicated by the fact that LCMV infection of previously immunized mice in which case no virus persists, also results in long-standing antigen specific CD8^+^ T-cell infiltration. This is unlike the situation for lung Trm, which require persistent antigen for long-term stability of Trm numbers (Uddback et al., manuscript in prep.), but consistent with findings from mice with VSV induced encephalitis (14).

Although this is not meant to be a vaccine study, our results clearly support the growing notion that local antigen represent a key to establish a focused immune response and durable protection. This understanding is relevant both in relation to strategies regarding prophylactic vaccination as well as in the setting of local tumor immunotherapy, where Trms also have been found to play a significant role (16, 67). Thus, local antigen delivery e.g., in the form of a non-replicating adenovector at the tumor site, may be of particular value when antigen expression on the tumor cells themselves is low.

At first sight, the critical need for local antigen as a precondition for establishing a Trm population in the CNS could be taken to represent a immunological conundrum, since it means that only infections which have previously invaded the CNS will have resulted in formation of stable local T-cell immunity. While this concept of immunity to repeated attacks with the same or closely related pathogens intuitively makes good sense in barrier tissues, it is more difficult to see the advantage in the CNS: only survivors of previous neuroinvasive infections will present an improved response, and if you are already able to survive on the basis of a primary response what then is critical advantage of an accelerated reactivity? However, in fact this argument could be equally applied to all infections, and perhaps simply ameliorating the clinical symptoms of infection and improving your physical capabilities may be critical for survival in the real world, where your capacity to avoid a predator or being able to catch a prey may make more difference, than the outcome of infection *per se*.

In addition to the implications for protection against infectious diseases, we also believe that our findings are relevant in relation to the understanding of autoimmunity in the CNS. Our results imply that primed circulating autoantigen specific T cells will accumulate in the CNS in significant numbers only if matching (auto)antigen can be accessed by the infiltrating cells. Under homeostatic conditions the initial recruitment is likely to be limited by the fact that there may not be the activated endothelium in the CNS required for significant extravasation of antigen primed circulating cells. However, in accordance with epidemiological data indicating a role for unrelated infections as triggers of MS (68), our results suggest that even unrelated CNS inflammation may provide access for a few cells with relevant specificity. These may then upon interaction with local antigen presenting cells further feed the inflammatory process and thus the formation of a pathogenic T-cell infiltrate. It is also implied that, once an autoreactive T-cell infiltrate is formed in the CNS, many of the recruited cells will be retained as functional Trms. If this assumption is extrapolated to humans, treatment of MS by targeting of circulating cells and prevention of their local extravasation, would be expected to be most effective early in the disease process, but less so during subsequent disease progression. In accordance with this prediction, the efficiency of an antibody like Natalizumab, which acts by preventing leukocyte extravasation has been noted to decrease with age (69).

In conclusion, we have shown that local antigen encounter is critical for establishment, but not maintenance of an antigen specific CD8^+^ T-cell memory population in the CNS, whereas inflammation in the absence of cognate antigen is only associated with limited and transient CD8^+^ T-cell infiltration. Thus, our results indicate that CD8^+^ Trms recovered from the CNS predominantly represent memories of previous immune responses to local infections and locally expressed autoantigens.

## Ethics Statement

Experiments were conducted in accordance with national Danish guidelines (Amendment # 1306 of November 23, 2007) regarding animal experiments as approved by the Danish Animal Experiments Inspectorate, Ministry of Justice, permission number 2015-15-0201-00623.

## Author Contributions

JC and AT: designed the study; AS, MF, and LN: performed the experiments and analyzed the data; AS, JC, and AT: interpreted the data; AS and AT: drafted the manuscript. All authors approved the final version.

### Conflict of Interest Statement

The authors declare that the research was conducted in the absence of any commercial or financial relationships that could be construed as a potential conflict of interest.
