# Supplementary material for: Local Antigen Encounter Is Essential for Establishing Persistent CD8+ T-Cell Memory in the CNS
Source: Front Immunol. 2019 Mar 4;10:351. doi: 10.3389/fimmu.2019.00351 (PMC6409353; doi:10.3389/fimmu.2019.00351)
Supplement: Supplementary file 1 [file Data_Sheet_1.pdf]

S1

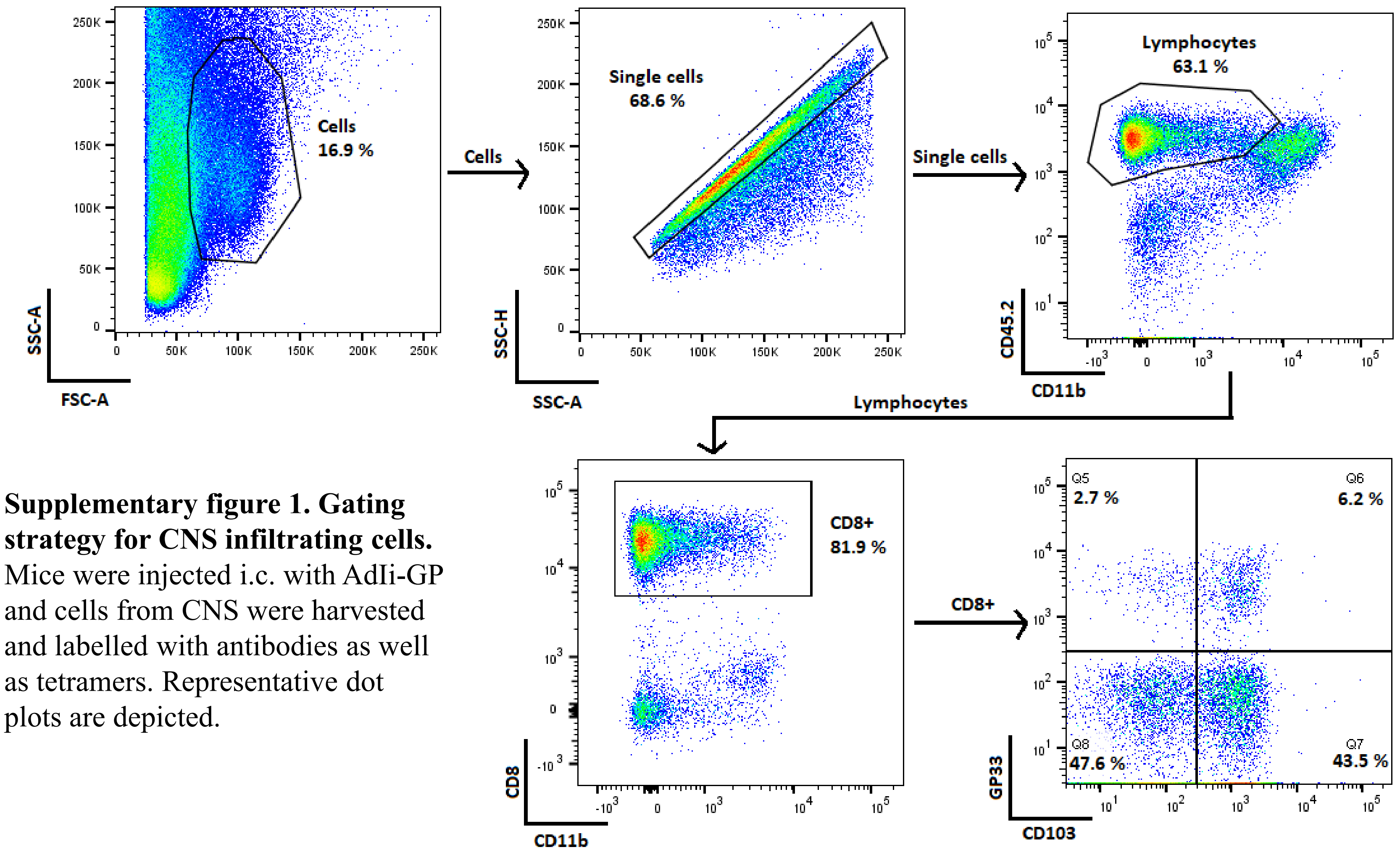

Supplementary figure 1. Gating strategy for CNS infiltrating cells. Mice were injected i.c. with AdIi-GP and cells from CNS were harvested and labelled with antibodies as well as tetramers. Representative dot plots are depicted.

S2

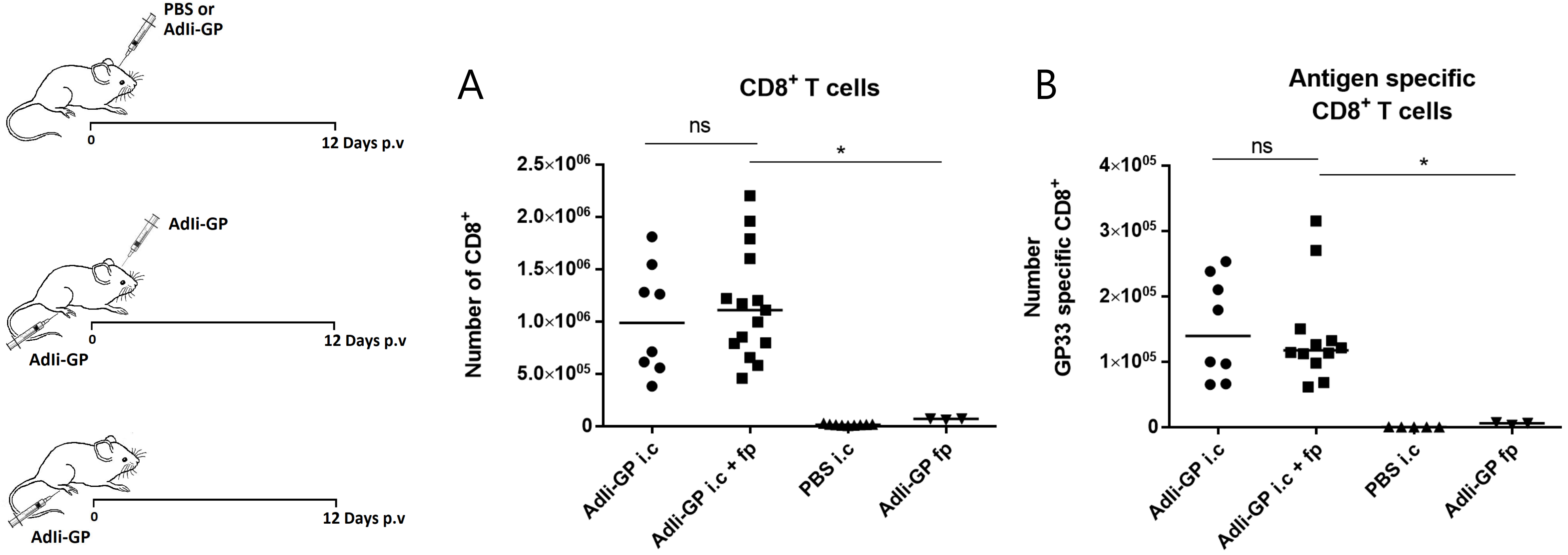

Supplementary figure 2. Local antigen inflammation is required for induction of a CD8$^+$ T-cell infiltrate in CNS. Groups of mice were injected with adenovectors or PBS as indicated. Twelve days later total numbers of infiltrating CD8$^+$ T cells (**A**) as well as numbers of antigen specific CD8$^+$ T cells in CNS (**B**) were enumerated. Symbols represent individual mice. Vertical bars represent group medians.

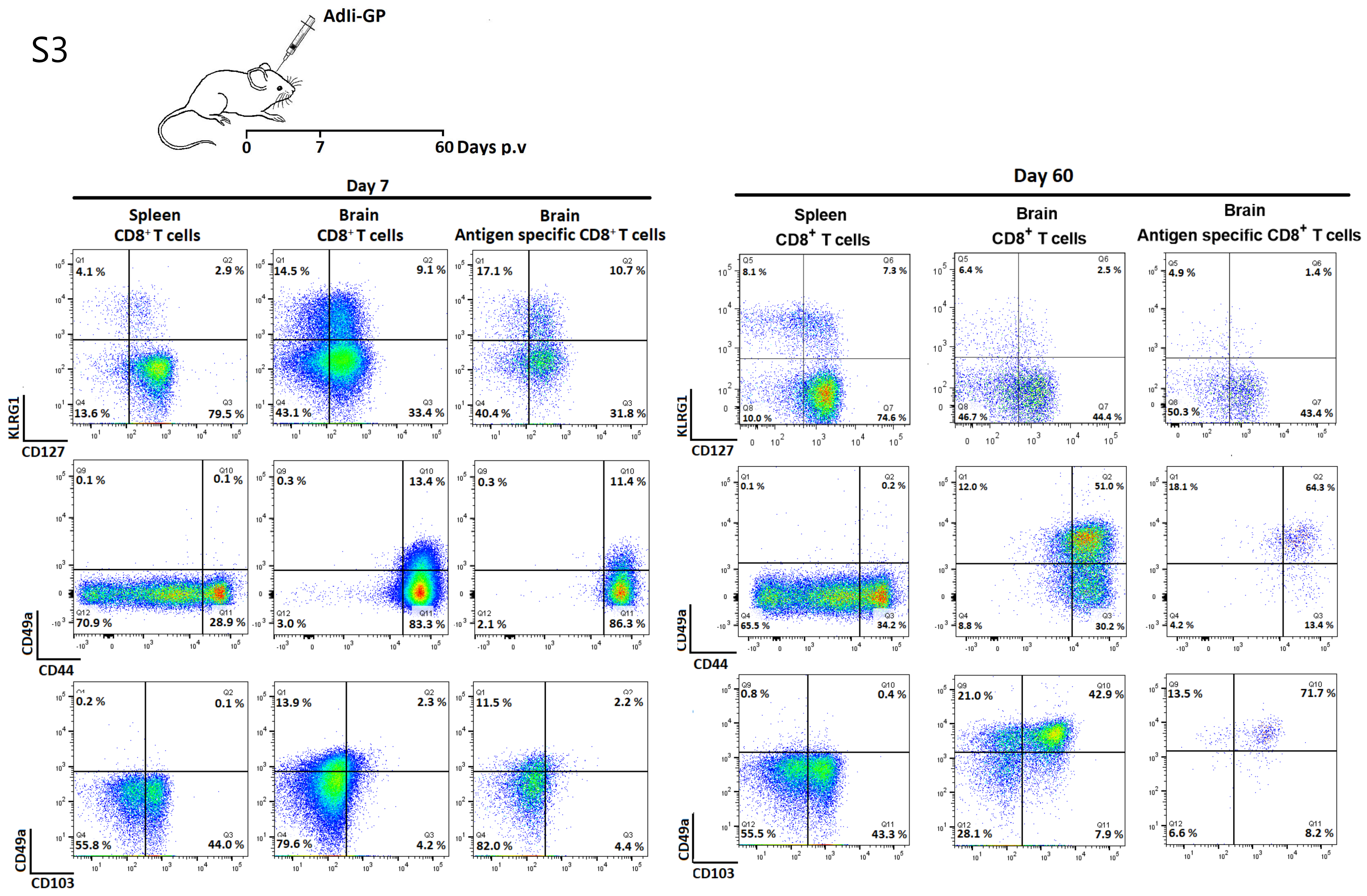

Supplementary figure 3. Phenotypic analysis of CNS infiltrating CD8^{+} T cells. Mice were injected i.c. with AdIi-GP. Seven and 60 days later, CD8^{+} T cells from CNS were harvested and analyzed for expression of Trm relevant cell surface markers. Representative plot of gated total CD8^{+} T cells (middle column) and antigen specific CD8^{+} T cells (right column); analysis of total CD8^{+} T cells from spleen (left column) have been included for comparison. Results from day 60 are representative of phenotypic analyses carried out on cells harvested between 12 and 103 days after i.c. inoculation.

S4

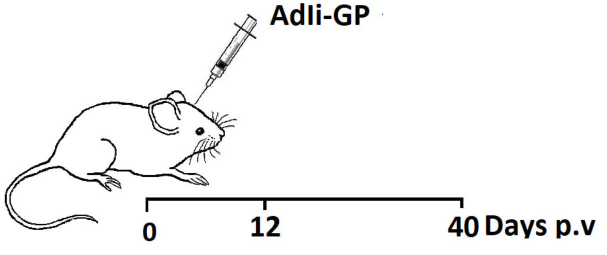

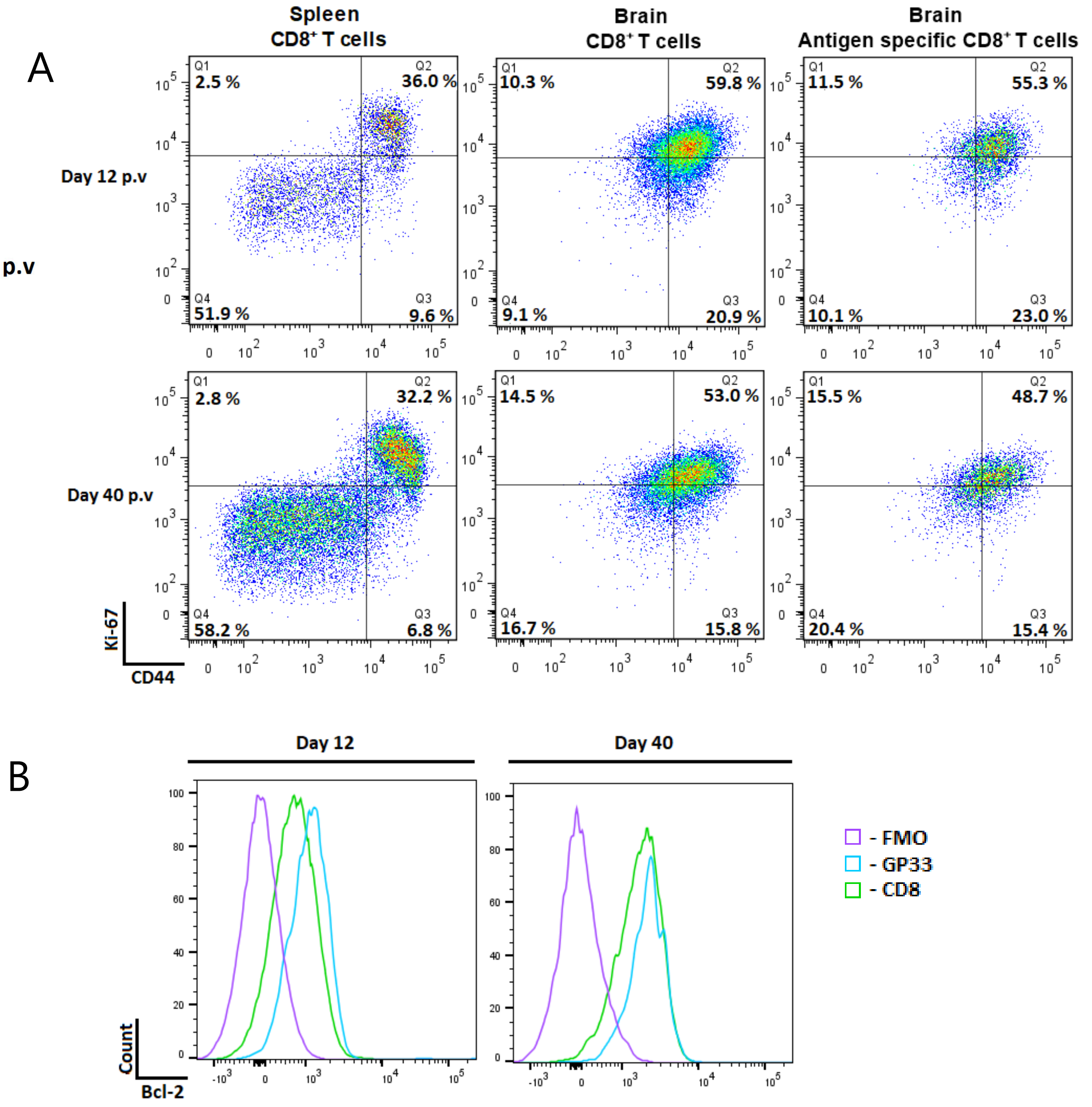

Supplementary figure 4. CNS infiltrating CD8$^+$ T cells express Bcl-2 and Ki-67. Mice were injected i.c. with AdIi-GP and on the indicated days, CD8$^+$ T cells from CNS were harvested and analyzed for expression of Ki-67 (**A**) and Bcl-2 (**B**). In (**A**) CD8$^+$ splenocytes from control animals were included to set the cut-off for Ki-67 expression. In (**B**) CD8 is representative of the total CD8$^+$ T population and GP33 refers to antigen specific CD8$^+$ T cells as determined by tetramers. FMO refers to level of expression in the presence of all included antibodies except anti-Bcl-2. The depicted results are representative of 4-5 mice per group in two independent experiments

S5

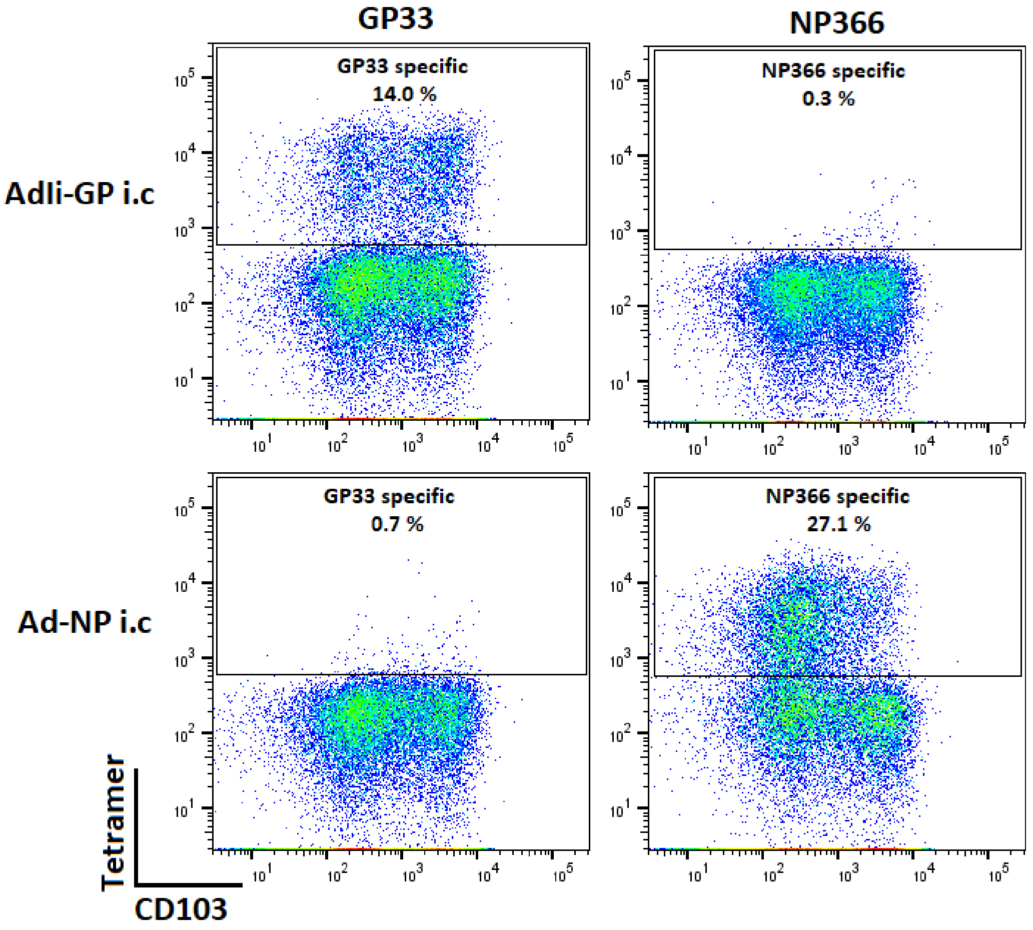

Supplementary figure 5. Validation of the antigen specificity of infiltrating $CD8^+$ T cells. Mice were injected i.c. with either AdIi-GP or Ad-NP , and 12 days later infiltrating cells were harvested from the CNS and analyzed for antigen specificity. Representative dot plots of gated $CD8^+$ T cells.

S6

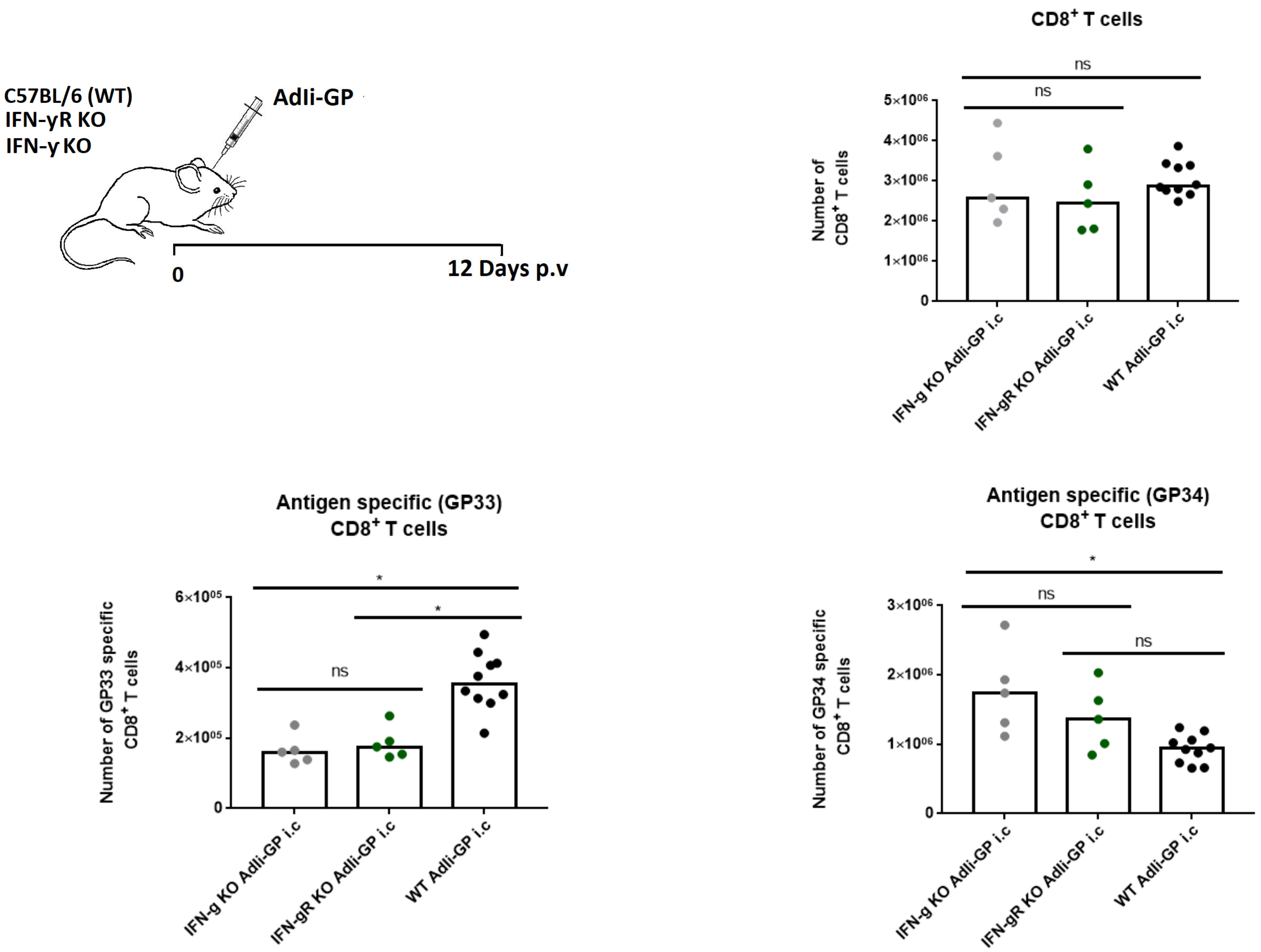

Supplementary figure 6. Role of IFN-γ in antigen driven CD8+ T-cell accumulation in CNS. Wild type mice and mice deficient in IFN-γ signaling were injected i.c. with AdIi-GP. Twelve days later infiltrating cells were harvested, and total number of CD8+ T cells together with numbers of antigen specific CD8+ T cells were determined. Symbols represent individual mice. Columns represent group medians.

S7

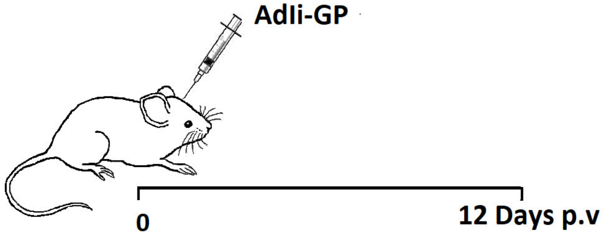

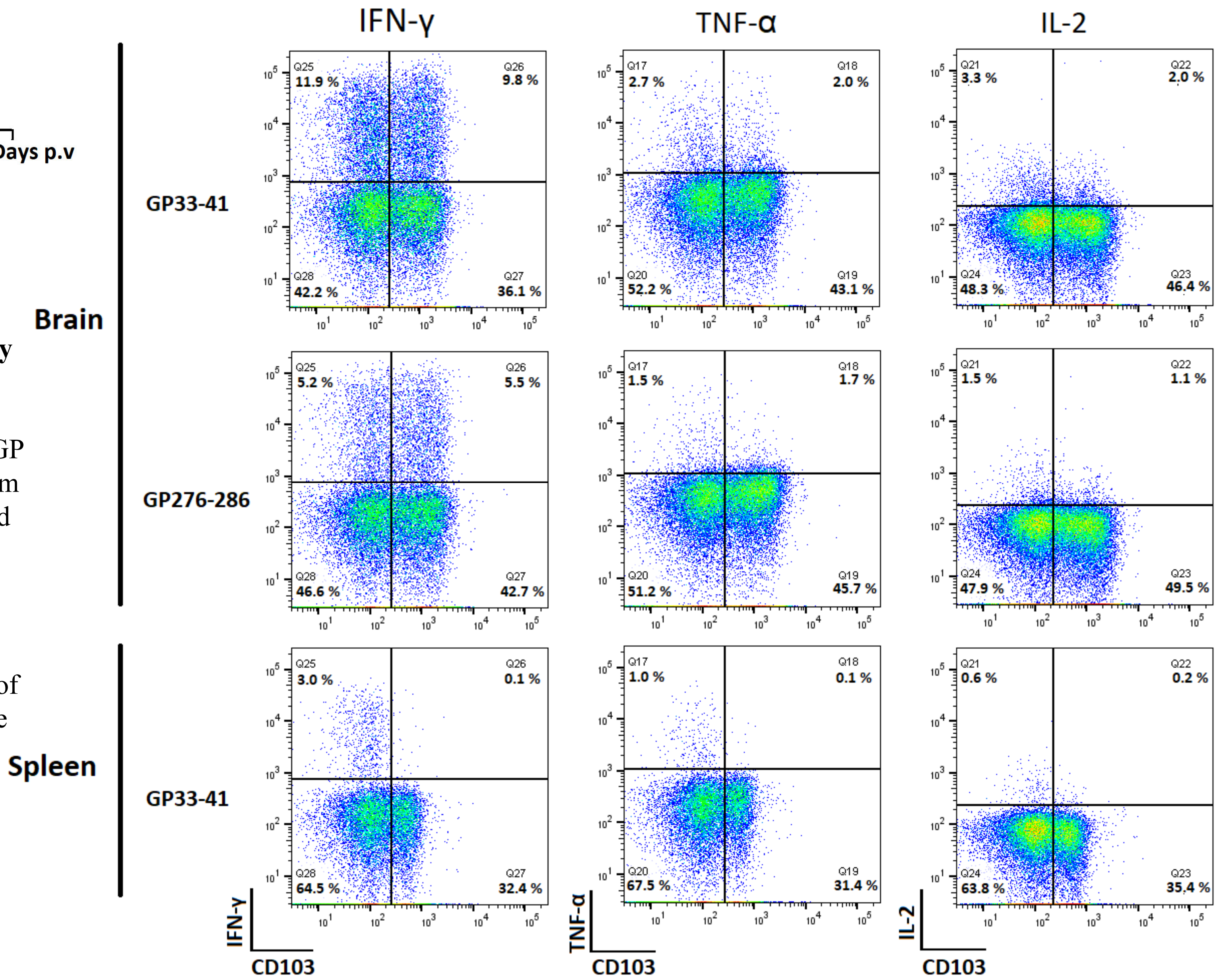

Supplementary figure 7. Capacity for cytokine productive as a function of CD103 expression. Mice were injected i.c. with AdIi-GP and 12 days later, $CD8^+$ T cells from CNS were harvested and stimulated with the indicated peptides. Following incubation cytokine production was evaluated using standard staining for intracellular cytokine. Dot plots representative of gated $CD8^+$ T cells from 5 mice are depicted.

S8

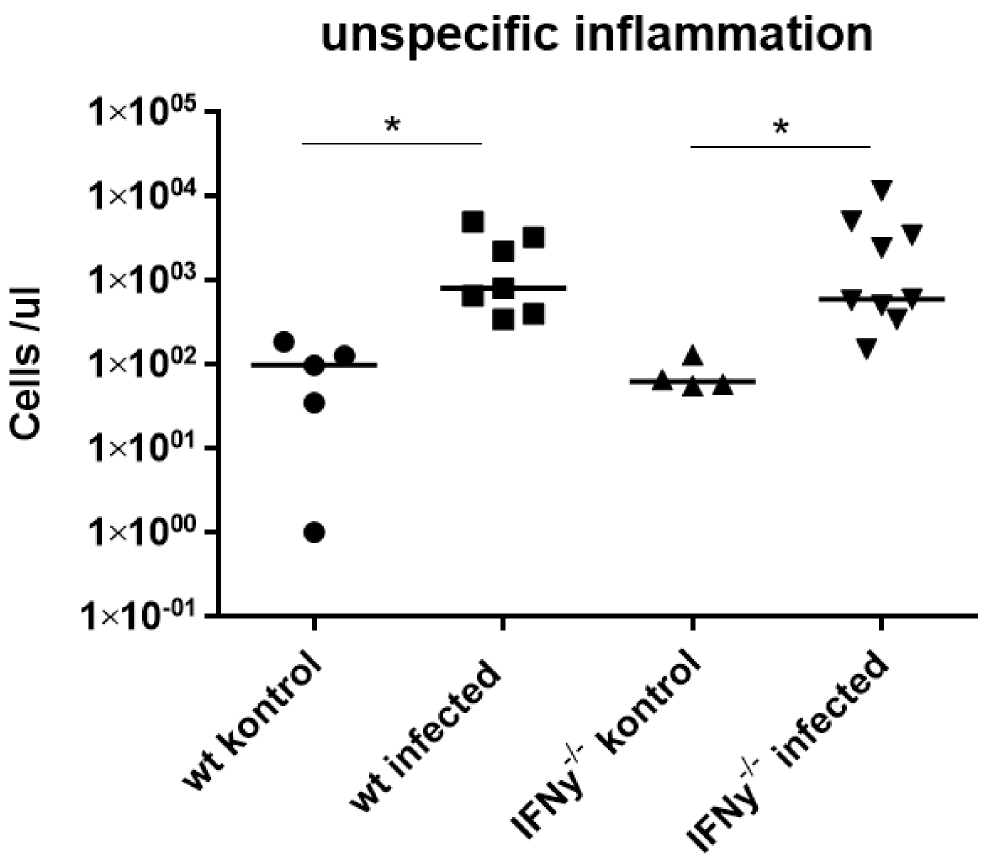

Supplementary figure 8. Virus-induced CSF infiltration in mice infected i.v. with LCMV. Wild type and IFN-γ KO mice were inoculated with LCMV i.v. Eight days later CSF was tapped, and numbers of recruited mononuclear cells were determined. Symbols represent individual mice. Vertical bars represent group medians.

S9

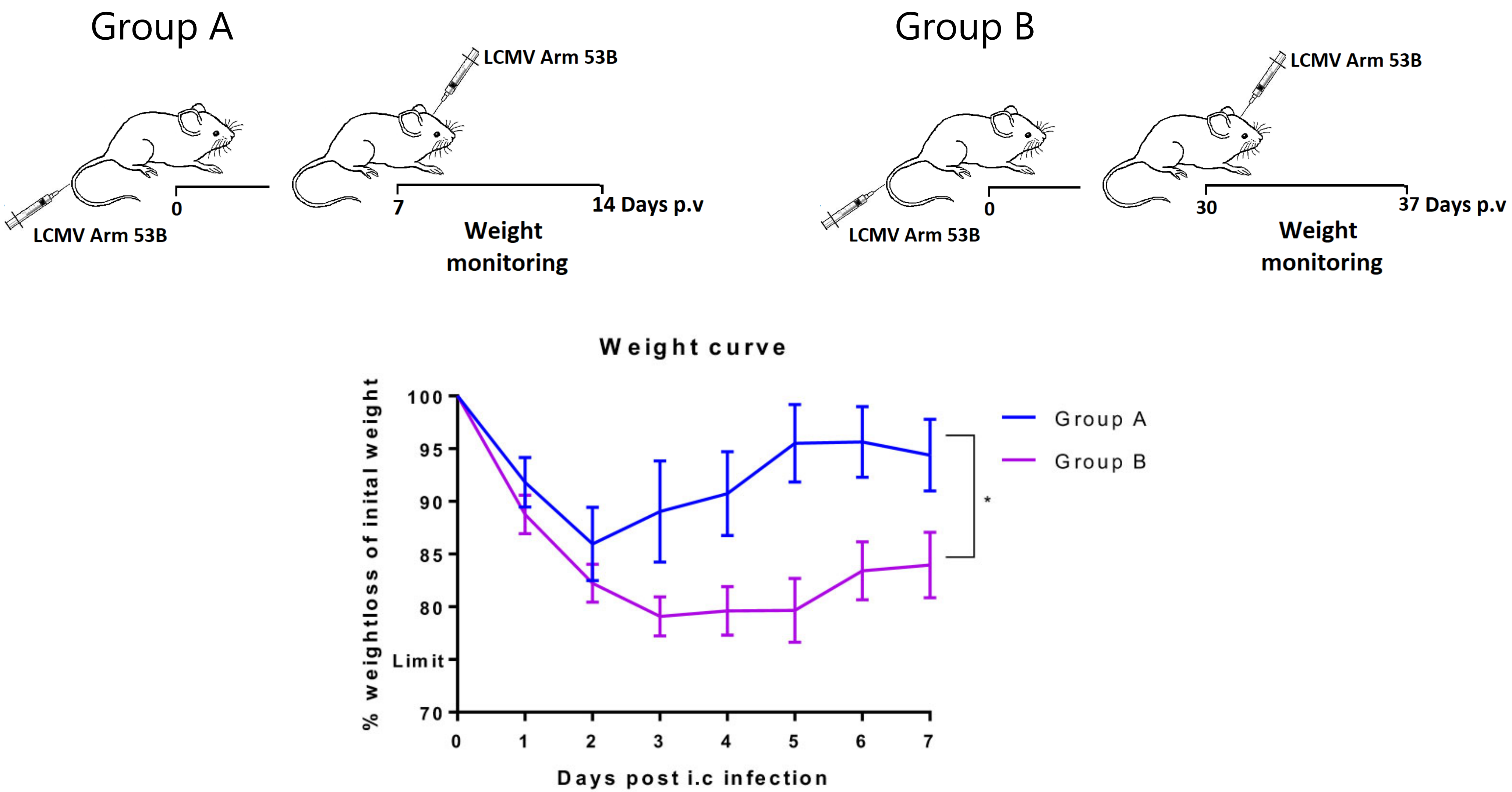

Supplementary figure 9. Severity of i.c. challenge as a function of time after initial i.v. priming. Wild type mice were inoculated with LCMV i.v. Seven or 30 days later mice were challenged i.c. with LCMV, and the weight loss associated with reinfection was followed. Means and standard deviations of groups of 10 mice are depicted.

S10

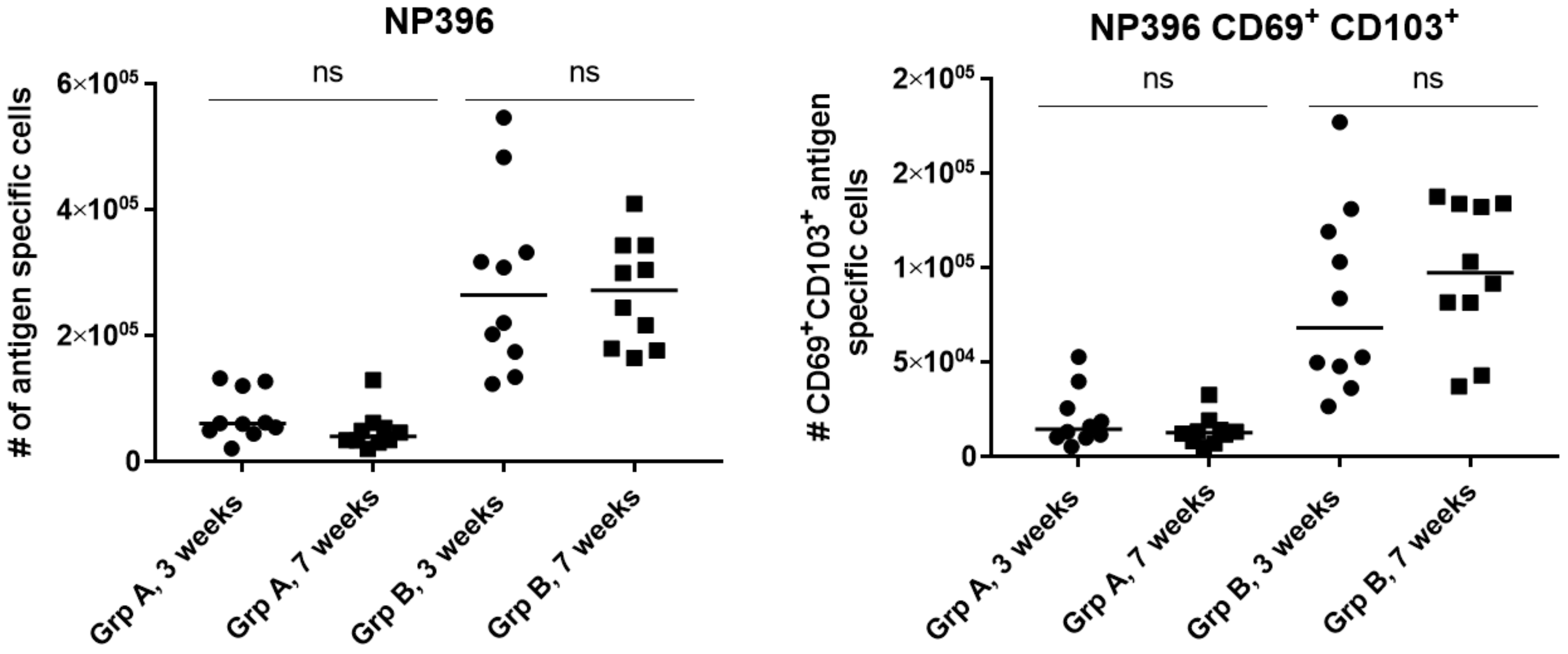

Supplementary figure 10. Long-term stability of the virus-induced $CD8^+$ T-cell infiltrate in CNS after transient re-infection. Mice were first infected with LCMV i.v. and subsequently challenged i.c. 7 (group A) or 30 (group B) days later. Three or 7 weeks after i.c. challenge, numbers of infiltrating $CD8^+$ T cells were determined. The results for 3 weeks are the same as depicted in fig.10 and have been included for comparison. Symbols represent individual mice. Vertical bars represent group medians.

S11

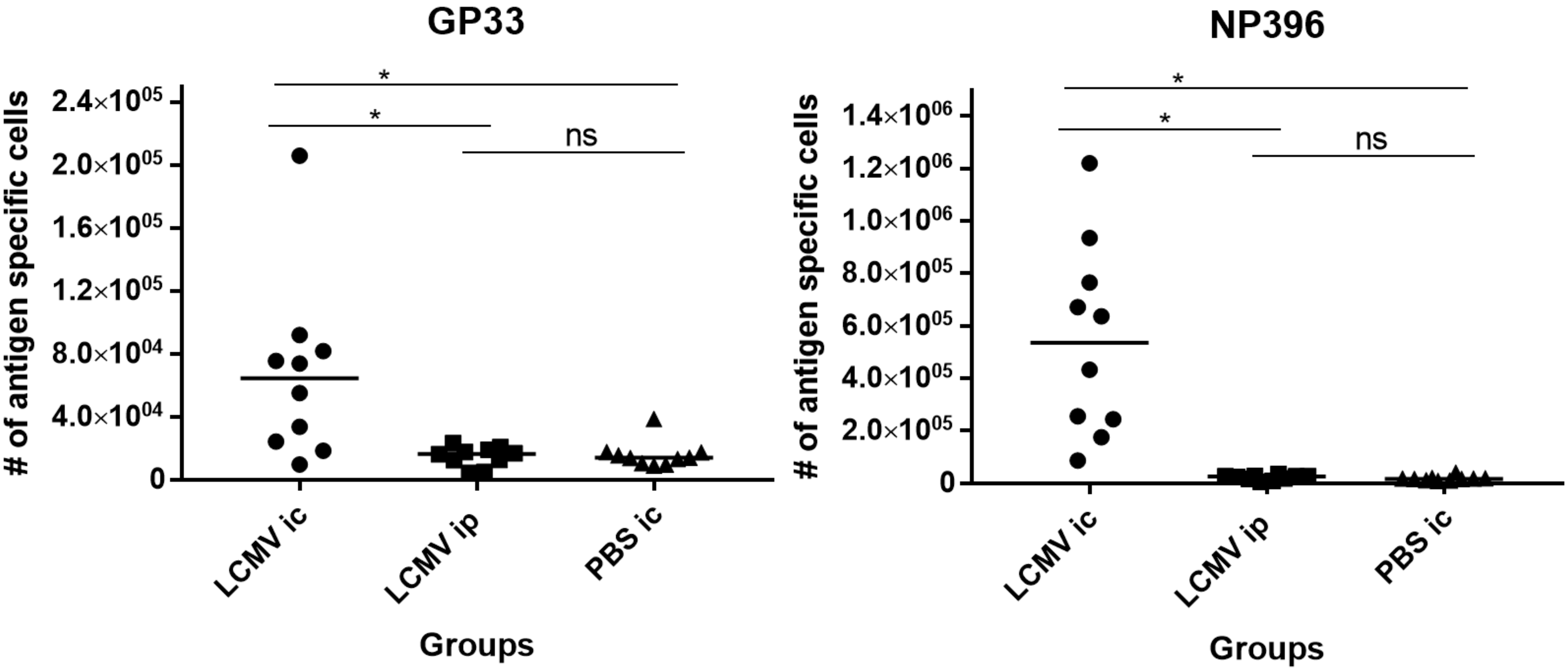

Supplementary figure 11. Failure of i.p. rechallenge to induce significant CNS infiltration. Mice were first infected with LCMV i.v. and subsequently challenged either i.c. or i.p. 30 days later. Three weeks after re-challenge, numbers of infiltrating $CD8^+$ T cells were determined. Symbols represent individual mice. Vertical bars represent group medians.
